# Sorting nexin 10 controls mTOR activation through regulating amino-acid metabolism in colorectal cancer

**DOI:** 10.1038/s41419-018-0719-2

**Published:** 2018-06-04

**Authors:** Yunchen Le, Sulin Zhang, Jiahui Ni, Yan You, Kejing Luo, Yunqiu Yu, Xiaoyan Shen

**Affiliations:** School of Pharmacy, Fudan University, Shanghai, 201203 PR China

## Abstract

Amino-acid metabolism plays a vital role in mammalian target of rapamycin (mTOR) signaling, which is the pivot in colorectal cancer (CRC). Upregulated chaperone-mediated autophagy (CMA) activity contributes to the regulation of metabolism in cancer cells. Previously, we found that sorting nexin 10 (SNX10) is a critical regulator in CMA activation. Here we investigated the role of SNX10 in regulating amino-acid metabolism and mTOR signaling pathway activation, as well as the impact on the tumor progression of mouse CRC. Our results showed that SNX10 deficiency promoted colorectal tumorigenesis in male FVB mice and CRC cell proliferation and survival. Metabolic pathway analysis of gas chromatography–mass spectrometry (GC-MS) data revealed unique changes of amino-acid metabolism by SNX10 deficiency. In HCT116 cells, SNX10 knockout resulted in the increase of CMA and mTOR activation, which could be abolished by chloroquine treatment or reversed by SNX10 overexpression. By small RNA interference (siRNA), we found that the activation of mTOR was dependent on lysosomal-associated membrane protein type-2A (LAMP-2A), which is a limiting factor of CMA. Similar results were also found in Caco-2 and SW480 cells. Ultra-high-performance liquid chromatography–quadrupole time of flight (UHPLC-QTOF) and GC-MS-based untargeted metabolomics revealed that 10 amino-acid metabolism in SNX10-deficient cells were significantly upregulated, which could be restored by LAMP-2A siRNA. All of these amino acids were previously reported to be involved in mTOR activation. In conclusion, this work revealed that SNX10 controls mTOR activation through regulating CMA-dependent amino-acid metabolism, which provides potential target and strategy for treating CRC.

## Introduction

Colorectal cancer (CRC) is the third most commonly diagnosed cancer in males and the second in females, with an estimated 1.4 million cases and 693 900 deaths occurring globally in 2012^1^. Current treatment approaches are not sufficient^2^. Only 40% of CRC are diagnosed at early localized stage and the 5-year survival is 13% when the disease has spread to distant organs^3^. The discovery of new therapeutic strategies in CRC treatment is an urgent need and has attracted a great deal of research interest^4–6^. Although cancer is widely known as a genetic disease, emerging evidence indicates that cancer is primarily a metabolic disease involving metabolism reprogramming such as Warburg effect^7^. Recently, metabolomics, an “omics” science aim to comprehensive profile metabolism in organism using cutting-edge analytical chemistry techniques and advanced computational methods, is being used to explore disease mechanisms and to identify novel drug targets^8^. In various bio-fluids and tissues of CRC patients, metabolomics studies have uncovered multiple altered metabolites in cellular respiration/carbohydrate, lipid, amino-acid, nucleotide, and other significant metabolite perturbations^9–11^. However, the understanding of the biological significance underlying the metabolism alternation is limited. Further study of the represented molecular mechanism of metabolism imbalance and its impact on CRC development will help to uncover new targets to treat CRC.

Sorting nexin 10 (SNX10) is one of the simplest structure members of sorting nexins (SNXs), a family of evolutionarily conserved proteins containing the phox-homology domain, which targets SNXs to endosome membrane through bind with phosphoinositide to regulate endosomal cargo sorting and trafficking^12^. An important way for the endosomes to participate in cell catabolism is lysosome-dependent autophagy. Autophagy has significant effects on cancer cell survival and may play dual role in carcinogenesis^13^. Chaperone-mediated autophagy (CMA), one of the three types of autophagy, is responsible to deliver the cytosolic proteins with a specific pentapeptide motif to be degraded in lysosomes^14^. The activation of CMA has been implicated in promoting lung cancer cell survival^15^. High level of CMA had been observed in large number of cancer cell lines and directly linked to tumor glycolysis, and pyruvate and fatty acid metabolism^16^. Thus, cancer cells may use CMA to rewrite the cellular metabolic homeostasis to facilitate tumor proliferation. Recently, we found that SNX10 controls CMA activity by mediating the maturation of cathepsin A, a key enzyme involved in lysosomal-associated membrane protein-2A (LAMP-2A) degradation, and plays an essential role in alcohol-induced liver injury and steatosis^17^. Through regulating chaperone-mediated autophagic degradation of p21^Cip1/WAF1^, SNX10 acts as a tumor suppressor in tumorigenesis and progression of colorectal cancer^18^. These results encouraged us to explore the possible role of SNX10 in metabolism reprogramming through modulating CMA activation. In the present study, ultra-high-performance liquid chromatography–quadrupole time of flight (UHPLC-QTOF)- and gas chromatography–mass spectrometry (GC-MS)-based untargeted metabolomics were used to analyze the metabolism alterations in the tumor tissues of wild-type (WT) and SNX10 knockout (KO) CRC male FVB mice, and the relation of amino-acid metabolism alteration and CRC cell proliferation and survival was investigated.

## Results

### SNX10 deficiency promotes colorectal tumorigenesis in male FVB mice

**Fig. 1:**
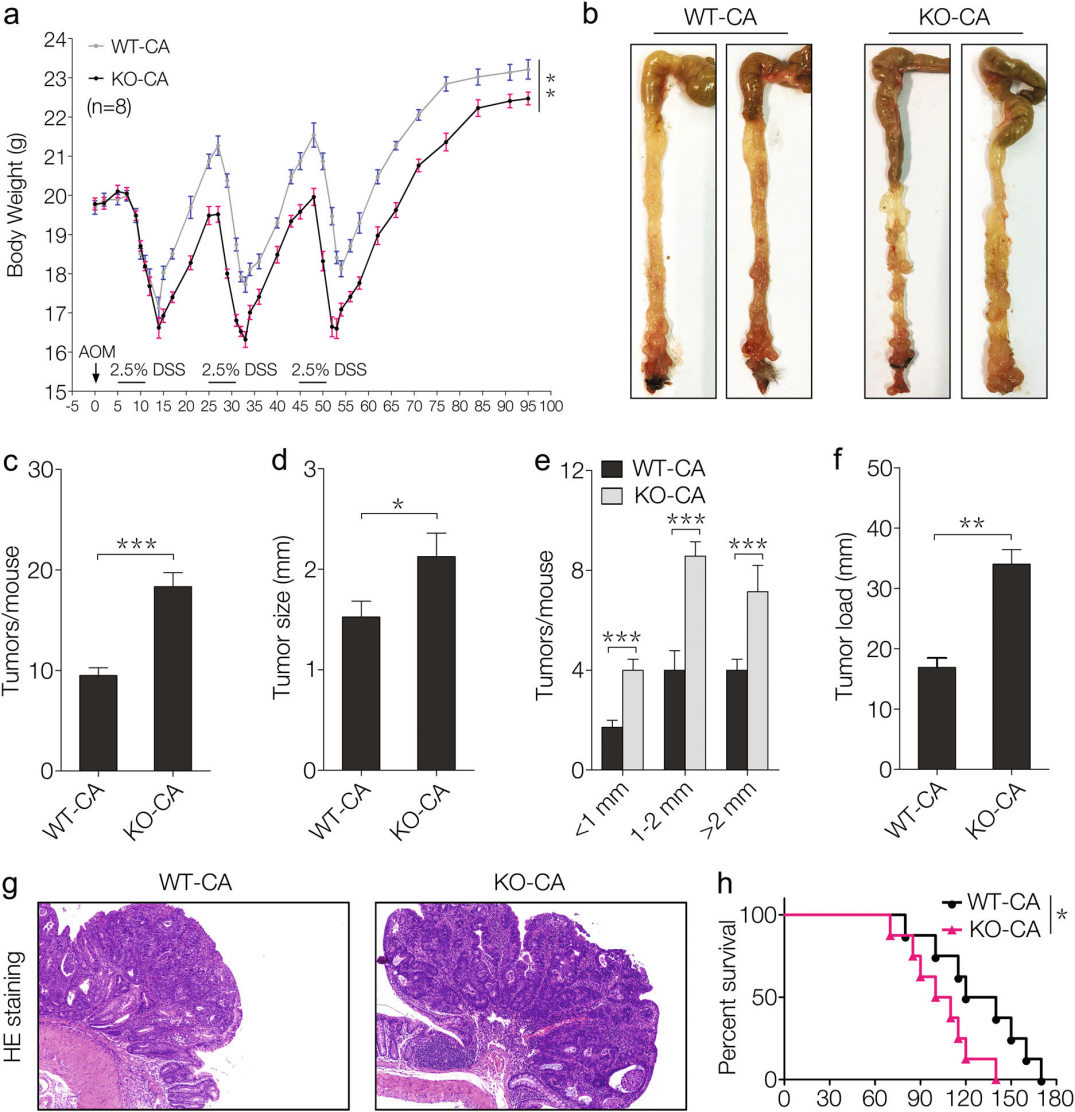
SNX10 deficiency promotes mouse colorectal tumorigenesis in male FVB mice. **a** Average body weight of WT and SNX10 KO CRC male mice were calculated. **b** Tumors inside the colorectum of WT and SNX10 KO CRC male mice were photographed. **c** Tumor numbers were counted. **d**, **e** Tumor diameter and distribution were measured. **f** The tumor load was determined by totaling the diameters of all tumors for a given mouse. **g** CRC tissues from WT and SNX10 KO mice were fixed and stained with H&E. **h** Survival curves of WT and SNX10 KO CRC male mice. The incidence of tumors was 100% in all mice, and the CRC mice were maintained with regular food and water until death. Survival curves were calculated according to the Kaplan–Meier method; survival analysis was performed using the log-rank test. *P < 0.05, **P < 0.01, and ***P < 0.001

To evaluate the possible role of SNX10 on tumorigenesis of CRC, we generated an azoxymethane (AOM)/dextran sulfate sodium (DSS)-induced CRC model in male FVB mice. SNX10 KO CRC mice had more weight loss and recovered more slowly during each DSS exposure compared with WT mice (Fig. 1a). The mice were sacrificed on day 95 following induction, the incidence of tumors was 100% in all mice. However, in SNX10 KO mice the number of tumors increased almost two times and the tumor sizes were larger compared to WT mice (Fig. 1b–e). Correspondingly, the tumor load, which represents the sum of the diameters of all tumors in a given mouse, was significantly increased in SNX10 KO mice (Fig. 1f). These results were also confirmed by histological assessment (Fig. 1g). Moreover, SNX10 KO CRC mice showed poorer survival (Fig. 1h). These results indicate that SNX10 deficiency significantly promoted tumorigenesis of CRC in male FVB mice.

### SNX10 deficiency facilitates HCT116 cell proliferation and survival under nutritional deprivation

**Fig. 2:**
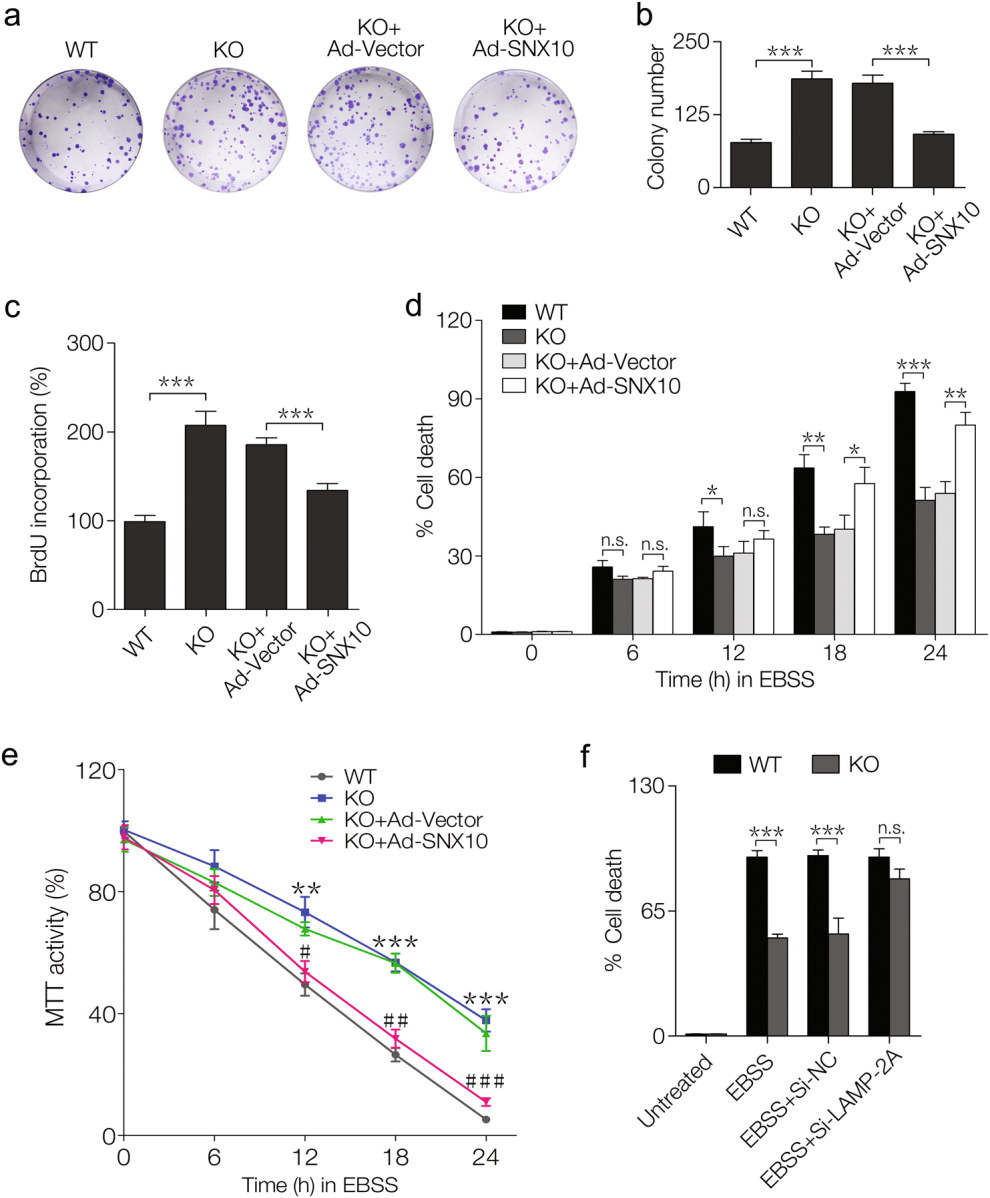
SNX10 deficiency facilitates HCT116 cell proliferation and survival under nutritional deprivation. **a** The colony-forming ability of WT, SXN10 KO HCT116 cells, and SXN10 KO HCT116 cells infected with Ad-vector or Ad-SNX10. **b** Quantification of colony numbers in **a**. **c** The proliferation of WT, SXN10 KO HCT116 cells, and SXN10 KO HCT116 cells infected with Ad-vector or Ad-SNX10 was measured by BrdU cell proliferation assay. **d**, **e** WT, SXN10 KO HCT116 cells, and SXN10 KO HCT116 cells infected with Ad-vector or Ad-SNX10 were cultured in EBSS for 0, 6, 12, 18, or 24 h. **d** The percentage of dead cells was determined by trypan blue exclusion assay at the indicated time points. **e** The cell viability was determined by MTT assay at the indicated time points. **f** WT and SXN10 KO HCT116 cells were untreated, or were treated with EBSS for 24 h, or were transfected with nontargeting siRNA (Si-NC) or LAMP-2A siRNA (Si-LAMP-2A), followed by EBSS treatment for 24 h, and the percentage of dead cells was determined by trypan blue exclusion assay. Data are derived from at least three independent experiments and represented as means ± SEM in the bar graphs. Not significant (n.s.), *P < 0.05, **P < 0.01, and ***P < 0.001

To further confirm the effect of SNX10 deficiency on CRC, we first measured the expression of SNX10 mRNA in NCM460 cell, which is a normal human colon mucosal epithelial cell line, and in three CRC cell lines HCT116, Caco-2, and SW480 cells (supplementary Fig. S1), and found that SNX10 mRNA level was decreased in CRC cells. We then compared the proliferation and survival ability between WT and SNX10 KO HCT116 cells. The colony numbers of SNX10 KO HCT116 cells were more than two times of WT cells, while SNX10 reintroduction could partially reversed this result (Fig. 2a, b). BrdU cell proliferation assay also showed that cell proliferation ability was enhanced by SNX10 deficiency and this effect was partially reversed by SNX10 reintroduction (Fig. 2c). To explore whether SNX10 is involved in CRC cell survival under nutritional deprivation, WT and SNX10 KO HCT116 cells were cultured in Earles balanced salt solution (EBSS). SNX10 KO cells showed more resistance to trypan blue staining than WT cells, and this effect could be partially reversed by SNX10 reintroduction (Fig. 2d), suggesting SNX10 deficiency facilitated CRC cell survival under nutritional deprivation. MTT assay also confirmed the impact of SNX10 deficiency on CRC cell survival under nutritional deprivation (Fig. 2e). Moreover, under nutritional deprivation, the difference of cell survival ability between WT and SNX10 KO HCT116 cells could be abolished by LAMP-2A depletion (Fig. 2f). LAMP-2A is the rate-limiting factor for CMA degradation. The level of LAMP-2A protein in the lysosomal membrane directly correlated with CMA activity. The results also indicate that SNX10 may be involved in CMA-mediated degradation. These results were validated in Caco-2 and SW480 cells (supplementary Fig. S2).

### Untargeted metabolomics reveals significant metabolism alternations in tumor tissues of CRC male mice caused by SNX10 KO

**Fig. 3:**
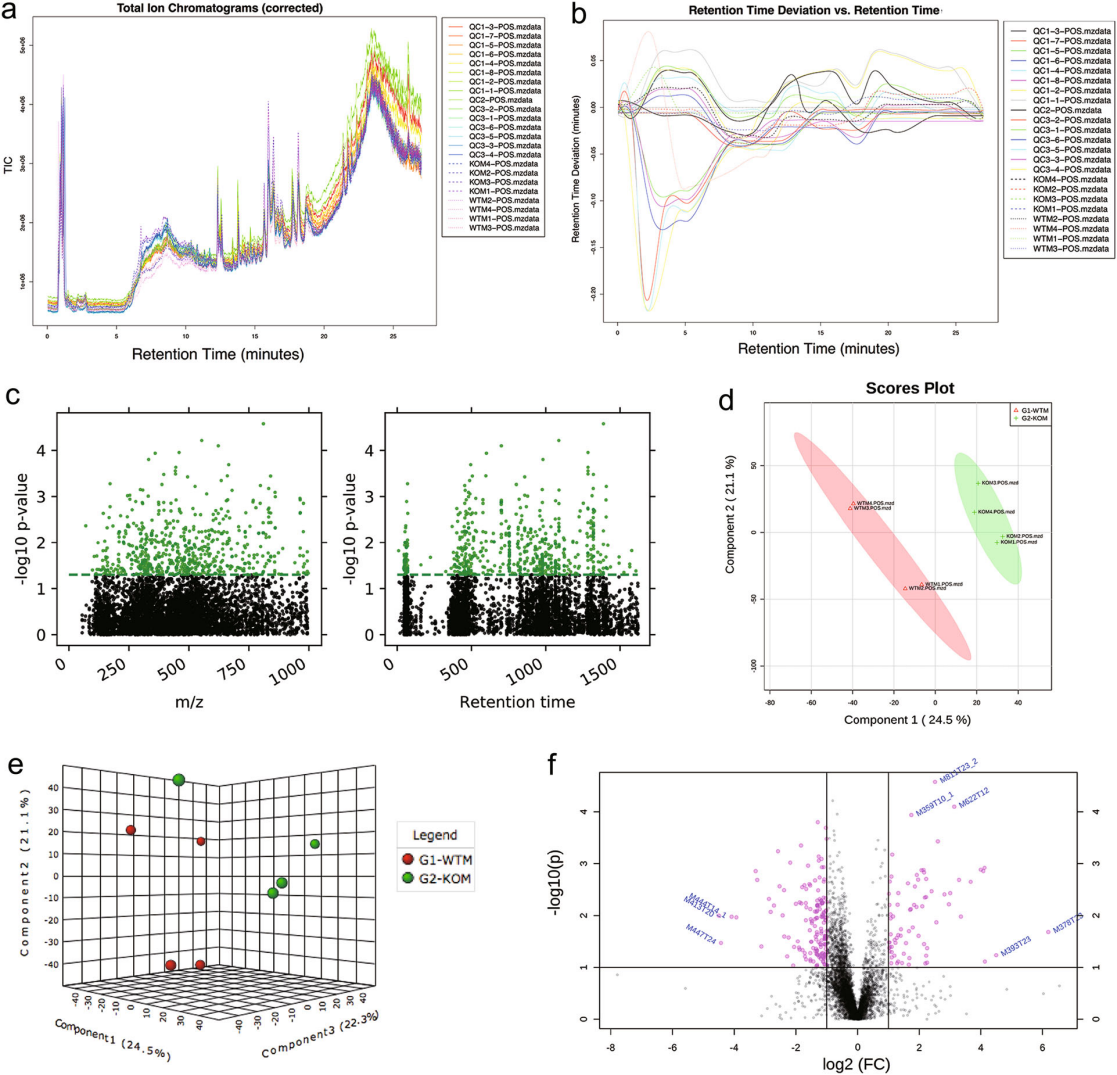
Untargeted metabolomics and multivariate statistical analysis of tumor tissues from CRC male mice using UHPLC-QTOF in positive ion mode disclosed significant metabolic alternations caused by SNX10 deficiency. **a** Total ion chromatograms of WT (WTM), SNX10 KO (KOM), and quality control samples (QC). **b** Retention time deviation of all samples. **c** Manhattan map showed the distribution of significant features (*P* < 0.05, green dots) in *m*/*z* and retention time. **d** 2D score plot between the selected PCs of PLS-DA model. **e** 3D score plot between the selected PCs of PCA model. **f** Volcano plot with fold change threshold and *t*-test threshold to select the important features

In order to investigate the metabolic alternations caused by SNX10 KO, untargeted metabolomics was applied on the colonic tumor tissues of male FVB mice. Liquid chromatography–MS (LC-MS)- and GC-MS-based analysis platforms were both used due to their different metabolite coverage. UHPLC-QTOF positive ion-mode analysis generated 8662 features (Fig. 3a). The retention time drifts of quality control (QC) samples were <0.2 min, which indicated good reproducibility of our methodology in this analysis mode (Fig. 3b). There were 835 positive ion features distributed in the *m*/*z* range from 50 to 1000 and retention time from 9.5 to 1619.7 s to be significantly changed (*P* < 0.05; Fig. 3c). Multivariable statistical analysis models were applied in order to obtain overview of the metabolome differences among the samples. Partial least squares-discriminant analysis (PLS-DA) score plot (Fig. 3d) and principal component analysis (PCA) model plot (Fig. 3e) provided the separation patterns among the most significant principal components. They showed a complete separation of WT and KO samples. Cross-validation bar graphs and details that used to evaluate the common performance of PLS-DA model were shown in supplementary Fig. S3. A total of 184 important features were selected by volcano plot (Fig. 3f) with fold change (FC) test threshold 2 and *t*-test threshold 0.05. The red dots represent features above the threshold. Both FCs and *P* values were log-transformed. The summary of features and metabolite numbers analyzed by untargeted metabolomics was shown in Table 1. The plots of negative ion-mode UHPLC-QTOF and GC-MS analysis were shown in supplementary Fig. S4-S5.

**Table 1:** The summary of features and metabolite numbers analyzed by untargeted metabolomics

	UHPLC-MS		GC-MS
	Positive ion mode	Negative ion mode	
Number of peaks in raw data	8662	2570	774
Number of peaks after data pre-processing	5140	1481	773
Number of significant features	835	437	117
Number of identified metabolites	34	50	24

### Carbohydrate, amino-acid, and nucleotide metabolism were upregulated by SNX10 deficiency

**Fig. 4:**
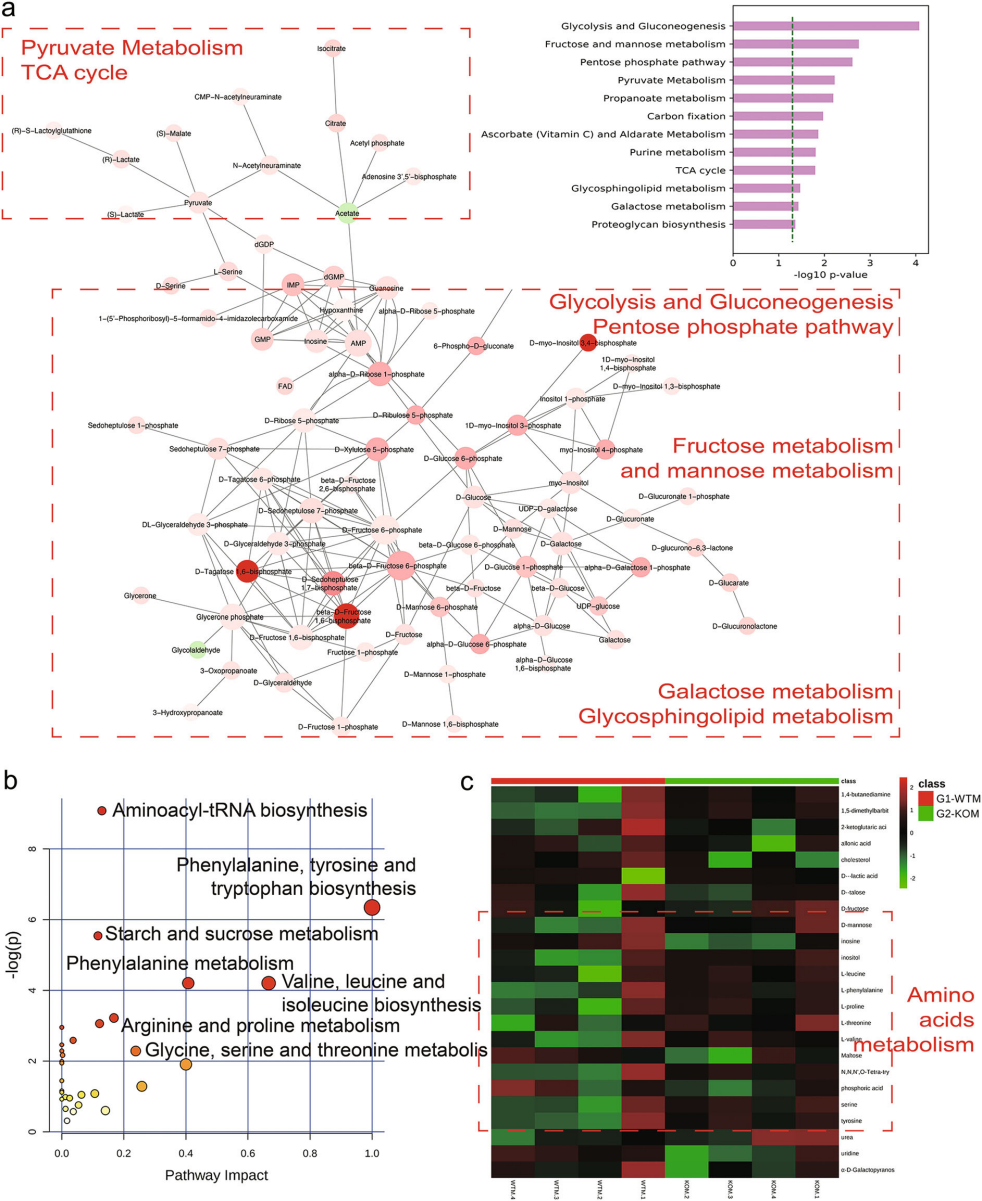
Metabolic pathway networks based on the untargeted metabolomics using UHPLC-QTOF in negative ion mode and GC-MS analysis. **a** Metabolic networks of activity pathways that based on the UHPLC-QTOF negative ion-mode analysis and generated from software Mummichog and visualized via Cytospace. Metabolites are colored green (concentration decrease) or red (concentration increase), the size and color intensity represent the connectivity of the metabolite in the networks and magnitude of fold change. **b** The metabolome view that based on the GC-MS analysis and generated from the web-based metabolomics analysis tool MetaboAnalyst. All matched pathways according to *P* values from pathway enrichment analysis and pathway impact values from pathway topology analysis. **c** Heatmap of the significant metabolites analyzed by GC-MS showed their concentration changing patterns across WT and KO samples

UHPLC-QTOF analysis raw data were processed to generate the active metabolic pathway networks (Fig. 4a). Negative ion-mode analysis demonstrated that carbohydrate, amino-acid, and nucleotide metabolism were upregulated. The active metabolic pathways included glycolysis, pyruvate metabolism, and tricarboxylic acid (TCA) cycle suggested that glucose uptake and biosynthesis using intermediate metabolites in TCA cycle were increased in SNX10 KO mice. Purine metabolism was upregulated accompanied by the active pentose phosphate pathway. Sharply elevated fructose and galactose metabolism in CRC indicate that they were important energy resources for the CRC tumor proliferation. Positive ion-mode analysis showed that purine metabolism, inositol phosphate metabolism, and starch metabolism were elevated in SNX10 KO tissues (supplementary Fig. S6).

Metabolic pathway analysis of GC-MS data revealed unique changes of amino-acid metabolism in SNX10-deficiency mice. A set of amino-acid metabolism and aminoacyl-tRNA biosynthesis were upregulated in SNX10 KO tissue samples (Fig. 4b). GC-MS data-based heatmap showed an important concentration change of metabolites in SNX10 KO mouse tissues compared to WT tissues (Fig. 4c). The red blocks represented increased concentrations of amino acids all associated with KO samples. All the metabolite information generated from untargeted metabolomics were listed in supplementary Table S1-S3. Among them, we paid special attention to the amino-acid metabolism because it has been closely connected with mTOR signaling activation and tumor development.

### Over-activation of CMA caused by SNX10 deficiency upregulates mTOR signaling activation

**Fig. 5:**
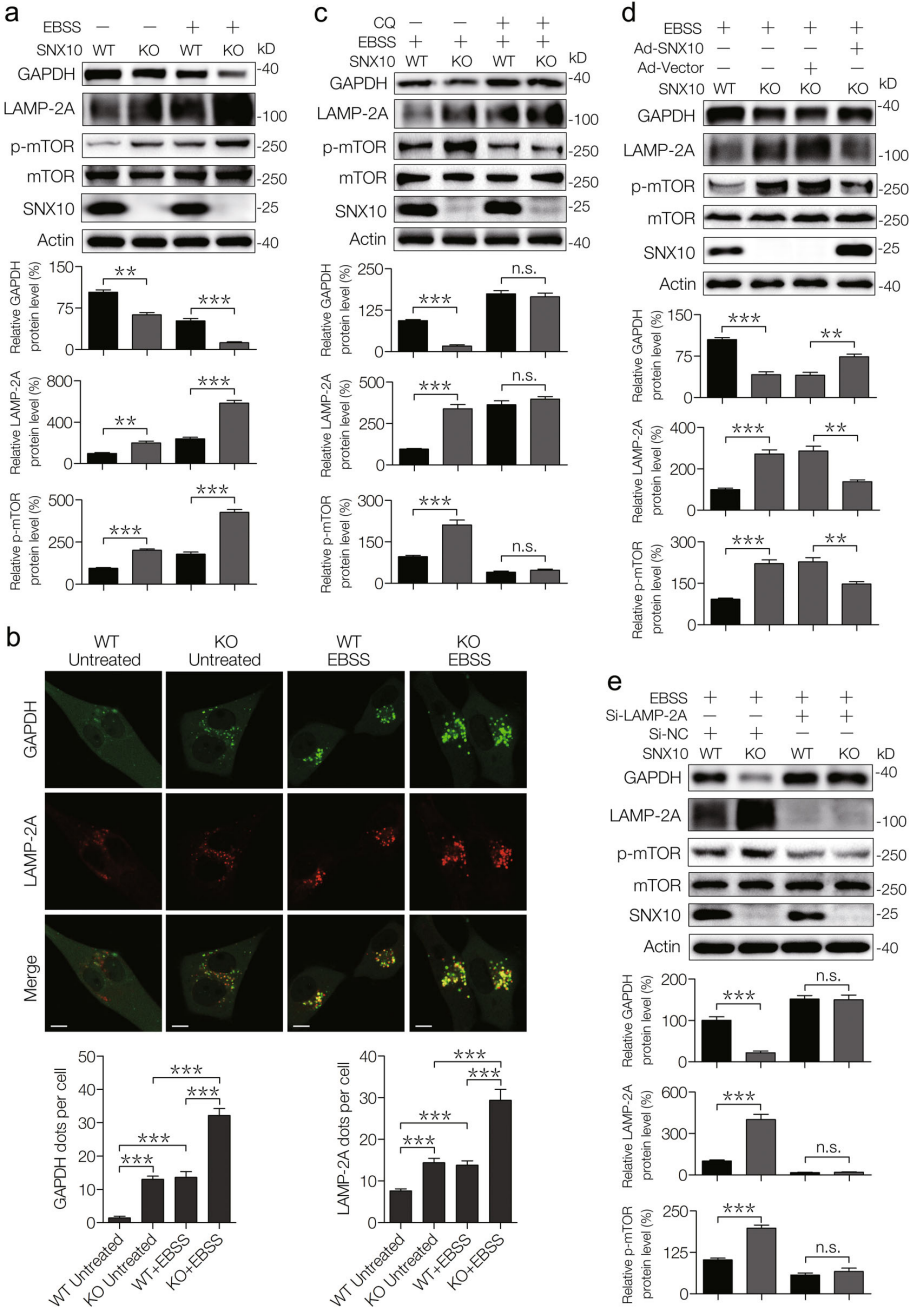
Persistent activation of CMA degradation caused by SNX10 deficiency activates mTOR signaling. **a** Representative western blots showed the expression of indicated proteins in WT and SNX10 KO HCT116 cells with the treatment of EBSS for 24 h. The bands of GAPDH, LAMP-2A, and p-mTOR were quantified using Quantity One software. **b** Co-staining of GAPDH and LAMP-2A in WT and SNX10 KO HCT116 cells treated with or without EBSS for 24 h. Anti-GAPDH and anti-LAMP-2A antibodies were used to detect endogenous GAPDH and LAMP-2A, respectively. Scale bar (5 μm). GAPDH- and LAMP-2A-positive puncta per cell from at least 200 cells in three independent experiments were quantified. **c** WT and SNX10 KO HCT116 cells were treated with EBSS and/or CQ for 24 h, the total proteins were isolated and immunoblotted with indicated antibodies. The bands of GAPDH, LAMP-2A, and p-mTOR were quantified using Quantity One software. **d** WT, SNX10 KO HCT116 cells, and SXN10 KO HCT116 cells infected with Ad-Vector or Ad-SNX10 were treated with EBSS for 24 h. LAMP-2A, GAPDH, and p-mTOR were detected by immunoblot, followed by densitometry quantification by Quantity One software. **e** WT and SNX10 KO HCT116 cells transfected with nontargeting siRNA (Si-NC) or LAMP-2A siRNA (Si-LAMP-2A) were treated with EBSS for 24 h, the total proteins were isolated and immunoblotted with indicated antibodies. The bands of GAPDH, LAMP-2A, and p-mTOR were quantified using Quantity One software. Data are derived from at least three independent experiments and represented as means ± SEM in the bar graphs. Not significant (n.s.), **P < 0.01, and ***P < 0.001

Due to the protein degradation through CMA can produce a large amount of cellular free amino acids, we then determined the possible correlation of increased CMA induced by SNX10 KO and amino-acid metabolism. LAMP-2A is an important limiting factor in CMA pathway. The expression of LAMP-2A in SNX10 KO cells increased dramatically under both normal and starvation conditions (Fig. 5a, b). Glyceraldehyde-3-phosphate dehydrogenase (GAPDH), a CMA substrate, negatively correlated with the expression of LAMP-2A and they highly overlapped in the cellular compartments (Fig. 5a, b). The upregulation of LAMP-2A and decrease of GAPDH in KO cells was suppressed by chloroquine (CQ), which is an autophagy inhibitor (Fig. 5c), or by SNX10 overexpression (Fig. 5d). The phosphorylated mTOR (p-mTOR) levels were upregulated by SNX10 deficiency and positively correlated with LAMP-2A (Fig. 5a, c); while decreased when SNX10 was reintroduced or LAMP-2A was interfered (Fig. 5d, e). These results indicate that CMA process was implicated in the mTOR activation induced by SNX10 deficiency.

### Cellular accumulation of amino acid caused by SNX10 KO was dependent on LAMP-2A

**Fig. 6:**
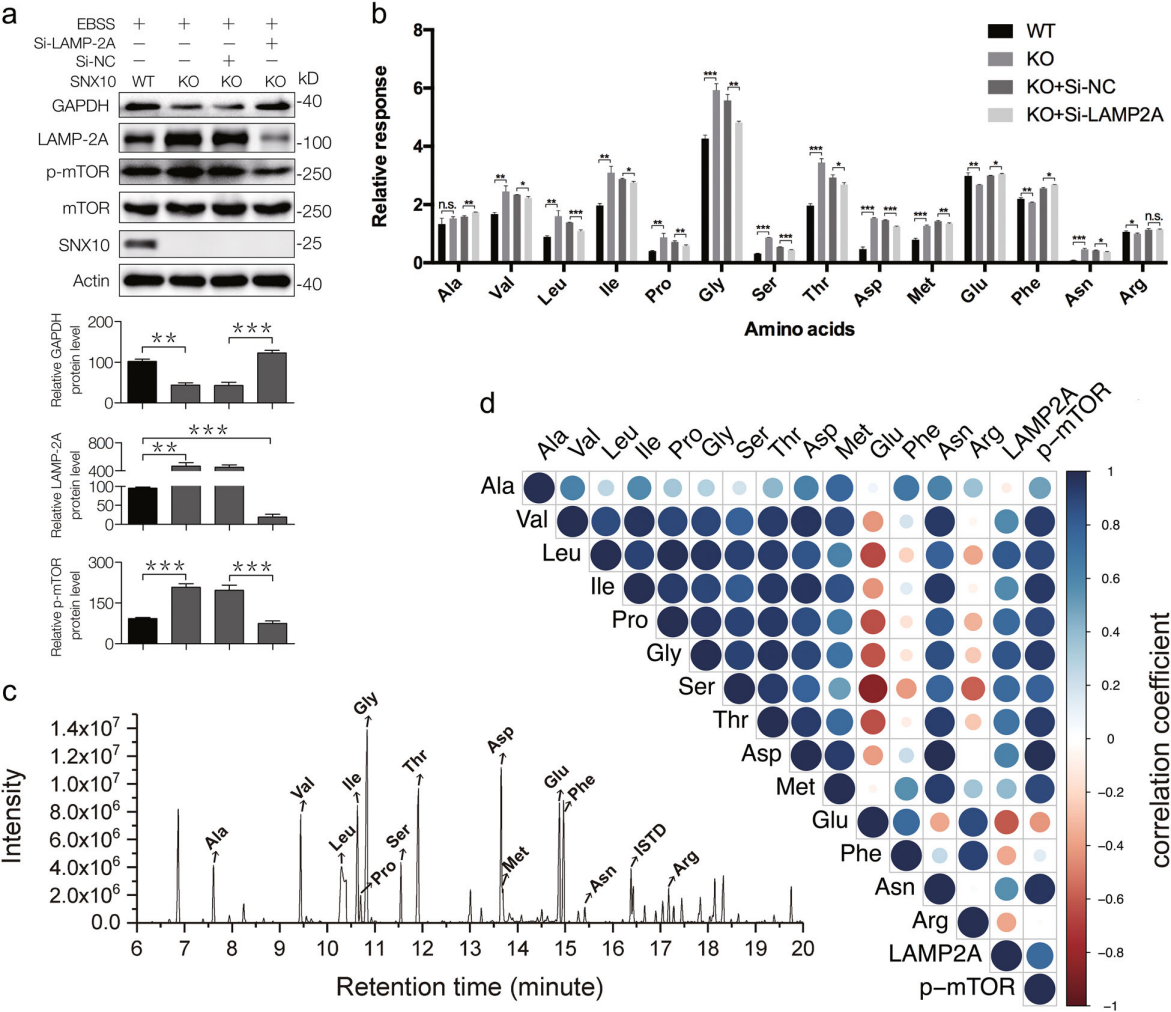
Targeted metabolomics analysis of amino acids using GC-MS validated that SNX10 deficiency enhanced CMA process leading to the accumulation of specific amino acids that can activate mTORC1 in CRC cells **a** WT, SNX10 KO HCT116 cells, and SNX10 KO HCT116 cells transfected with nontargeting siRNA (Si-NC) or LAMP-2A siRNA (Si-LAMP-2A) were treated with EBSS for 24 h, the total proteins were isolated and immunobloted with indicated antibodies. The bands of GAPDH, LAMP-2A, and p-mTOR were quantified using Quantity One software. **b** Relative levels of amino acids in WT, SNX10 KO and transfected with nontargeting siRNA (Si-NC) or LAMP-2A siRNA (Si-LAMP-2A) HCT116 cells. **c** Represented chromatogram of targeted metabolomics analysis of amino acids using GC-MS. **d** Pearson correlation analysis of amino-acid cellular concentrations with LAMP-2A and p-mTOR levels, blue dots represent positive correlation and red dots represent negative correlation, the deeper the color of the dots the greater the radius and the stronger the correlation. Data are derived from at least three independent experiments and represented as means ± SEM in the bar graphs. Not significant (n.s.), *P < 0.05, **P < 0.01, and ***P < 0.001

To confirm the relationship of CMA and mTOR activation, LAMP-2A siRNA was used to impair CMA activity. As shown in Fig. 6a, p-mTOR levels were elevated in SNX10 KO cells and decreased when LAMP-2A was interfered. Selective ion mode GC-MS-based targeted metabolomics was used to measure relative concentrations of 14 amino acids, which were observed in untargeted metabolomics or identified as important mTORC1 activators in previous study in WT, KO, and LAMP-2A siRNA cells (Fig. 6a)^19^. The results showed that 13 amino acids, including Val, Leu, Ile, Pro, Gly, Ser, Thr, Asp, Met, Asn, Glu, Phe, and Arg were changed by SNX10 KO and most of them were increased except Glu, Phe, and Arg (Fig. 6b). After LAMP-2A siRNA, the elevated amino acids induced by SNX10 KO were decreased. The represented chromatograph of these amino acids was shown in Fig. 6c. We then analyzed the Pearson correlation coefficients between these amino acids and levels of LAMP-2A and p-mTOR. The results showed that the concentration of Val, Leu, Ile, Pro, Gly, Ser, Thr, Asp, Met, and Asn had great correlation with LAMP-2A and p-mTOR (Fig. 6d).

The relative values of amino-acid concentrations were obtained by comparison with internal standard (ISTD) tridecanoic acid. The added accurate weight of tridecanoic acid was 24 μg for each sample. Results showed that glycine had the highest cellular level while asparagine concentration was the lowest among the analyzed amino acids. Due to the different ionization levels and response factors of these amino acids, the absolute cellular concentrations of these amino acids could not be directly calculated by the relative coefficient. Nevertheless, these results are sufficient to illustrate the amino-acid concentration differences between groups.

## Discussion

In the present study, we investigated the comprehensive metabolic alternations in SNX10-deficient CRC male FVB mice using untargeted metabolomics analysis, and explored its regulatory mechanism underlying increased amino-acid metabolism in SNX10 KO cells and its impact on CRC cell proliferation and survival. Our metabolomics characterization of SNX10 KO CRC male mice is consistent with the hallmarks of cancer metabolism^20^, suggesting that the accumulative amino acids caused by SNX10 KO have profound effect on the CRC cell growth and differentiation. Our study also demonstrates that over-activated CMA by SNX10 deficiency was responsible for the upregulated amino-acid metabolism and mTOR signaling activation; eventually promoted the abnormal growth and proliferation of tumor cells.

mTOR signaling can integrate signals from growth factors, nutrients, mutagens, and hormones to induce cell proliferation, resistance to apoptosis, and autophagy^21^. It is frequently dysregulated in a variety of human cancers, including colorectal cancer^22^. K-RAS or PIK3CA mutation could activate mTOR signaling and promote CRC cell growth^23^. Clinically, mTOR manifests poor prognosis in stage II CRC patients^24^. Inhibition of mTOR expression results in decreased human CRC cell growth in vitro and in vivo^25^. Therefore, mTOR represent an attractive antitumor target in combination with strategies to target other pathways in CRC^26^.

Multiple upstream signaling inputs including amino acid participate in the regulation of mTOR activation^27^. mTOR comprises two complexes, termed mTORC1 and mTORC2. Only mTORC1 is controlled by metabolites, such as glucose and amino acid^28^. Lysosomes are key sites for mTORC1 activation that regulated by the small guanosine triphosphatases (GTPase) Rheb and Rag, which reside at the lysosomal surface where they function as regulator of the mTORC1 kinase activity^29^. A key event in amino-acid signaling is the Rag GTPase-mediated translocation of mTORC1 to the surface of lysosomes. Lysosomes sense amino-acid signaling via vacuolar H(+)-adenosine triphosphatase (V-ATPase) to activate mTORC1, which is known as the “inside-out” mechanism^30^. Amino acids promote Rag to bind to Raptor (regulatory associated protein of mTOR) subunit of mTORC1 with consequent mTORC1 recruitment to lysosomes for its phosphorylation^31^. On the other hand, the dynamic of lysosomal amino acids is mTOR-dependent. Inhibition of mTOR strongly reduced the lysosomal efflux of most essential amino acids in nutrition starvation^32^. Due to its endosomal sorting and signaling functions, we consider SNX10 could affect the activation process of amino-acid metabolites to mTOR signaling by regulating lysosome-dependent processes, such as CMA.

Our recent study demonstrated SNX10 deficiency impaired the maturation of cathepsin A, which is a key enzyme involved in LAMP-2A degradation, thus increased CMA activity^17^. In the present study, we also confirmed that SNX10 KO upregulated LAMP-2A and CMA activity. CMA has high correlation with cancers. In contrast to the inhibition of macroautophagy in malignancy, upregulated CMA is required for cancer cell proliferation^33^. The interplay of CMA and macroautophagy has been reported in the literature. Induction of CMA compensates for the impairment of macroautophagy to promote hepatocellular carcinoma cell survival in the cirrhotic liver^34^. CMA-mediated anti-macroautophagic property is a key feature of tumorigenesis and metastasis in breast cancer^35^. Our recent study also showed that SNX10 KO inhibited macroautophagy, while increased CMA activity, promoting tumorigenesis and progression of colorectal cancer in male mice^18^. The connections between the metabolic changes and the development of cancer caused by CMA process is attracting more and more attention. CMA substrate proteins involved in the glycolysis, which contributes to the Warburg effect in cancer cells^36^. Metabolomics showed distinct effects of CMA and metabolic dysfunction, including increased branched-chain amino-acid levels, including Leu, Val, and Ile, in mice muscle and heart during aging^37^. CMA is also affected by ketone bodies, caloric restriction, and exercise in the context of neurodegenerative diseases^38^. Therefore, the influence of CMA on disease development is closely related to its role in cell catabolism and anabolism. However, to what extent CMA impact cell metabolism to promote CRC onset and progression remains largely unclear. CMA contributes to cellular QC through the removal of damaged or malfunctioning proteins^39^. Consequently, CMA has the potential to reshape the cell metabolism to promote cancer development. The elevated amino acids are not only the results of CMA substrate protein degradation but also act as cell signaling transductor to support tumorigenesis.

Given the close relationship between CMA and disease, its regulatory mechanism has aroused the interest of researchers. CMA process is mediated by binding to heat shock-cognate chaperone of 70 kDa (HSC70)^40^. Then LAMP-2A is required to bind to the substrate proteins in lysosomes before the translocation can occur^41^. The substrate protein is ultimately degraded into amino acid by lysosome hydrolytic enzymes. Levels of LAMP-2A at the lysosomal membrane are limiting factor for CMA^42^ since it helps the lysosomal uptake of approximately 30% modified and oxidatively damaged soluble cytosolic proteins to transfer directly into the lumen of lysosomes for degradation^43–45^. Recently, LAMP-2A was found to be coupled with several diseases to CMA pathway, such as breast cancer^46^, Parkinson disease^47^, and hepatocellular carcinoma^48^. Endosomes and lysosomes coordinate a balance of cellular materials for key cell processes. The latest research showed that endoplasmic reticulum stressors lead to directly phosphorylation of LAMP-2A and CMA activation^49^. The present study confirmes that SNX10 deficiency enhanced CMA through the LAMP-2A mediated signaling in CRC tumor cells.

**Fig. 7:**
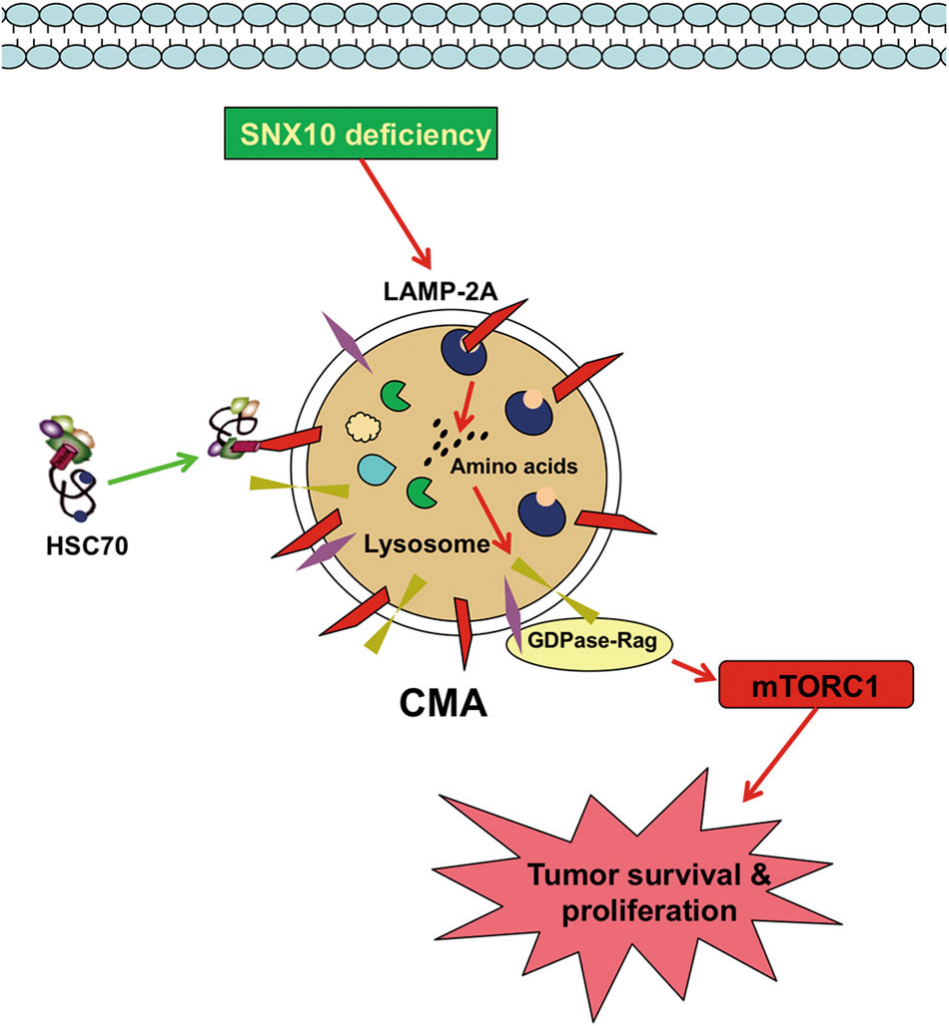
Hypothetical regulatory mechanism of SNX10 on amino-acid metabolism through CMA pathway and its effect on tumor progression by mTORC1 activation

Present targeted metabolomics study on CRC cells revealed that CMA selectively upregulated the levels of amino acids that are indispensable for the activation of mTORC1. The levels of Val, Leu, Ile, Pro, Gly, Ser, Thr, Asp, Met, and Asn sharply increased in SNX10 KO cells compared with WT cells, thereafter decreased when CMA pathway was partially suppressed. This result suggests that CMA at least partially contributed to these specific amino-acid accumulation caused by SNX10 deficiency. Notably, all of these amino acids are essential for the activation of mTORC1 according to the literature^19^. The mechanisms of the amino-acid sensors and recognition in mTORC1 activation are still being identified. Several studies have explored the selectively regulating mechanism of mTOR activation by amino acids, especially Leu, Arg, and Gln, which are the most important amino acids in mTORC1 activation. Human member 9 of the solute carrier family 38 (SLC38A9) was proposed as a lysosomal membrane-resident protein competent of the amino-acid sensing machinery that controls the activation of mTORC1^50^. SLC38A9 signals Arg sufficiency to mTORC1 activation^51^. Gln flux regulates mTOR under a mechanism of bidirectional transporter SLC7A5/SLC3A2^52^. Study found that the Leu-binding capacity of Sestrin 2 is required for Leu to activate mTORC1 in cells^53^. Therefore, the amino-acid regulatory mechanism of mTORC1 is different. In our study, instead of Arg and Gln, we found a group of amino acids that represented by Leu, Val, and Ile was closely correlated with SNX10/CMA/mTOR signaling. Whereas Arg showed no close correlation with mTOR phosphorylation, Glu and Phe even negatively connected with p-mTOR. Their concentrations were also decreased in SNX10 KO cells compared with WT cells. These results indicate that CMA process may be the unique mechanism on amino-acid-mediated mTORC1 activation in SNX10-deficient cells. Our data suggest that SNX10 deficiency enhanced CMA-mediated degradation of targeted proteins to increase specific amino acids, and subsequently activated mTORC1 to facilitate the tumor progression (Fig. 7).

In conclusion, our study showed that SNX10/CMA/amino acid/mTOR axis promoted CRC progression in male FVB mice. SNX10 could regulate the amino-acid metabolism of CRC cells by controlling CMA pathway. The upregulated cellular specific amino acids activated mTORC1 to promote CRC tumorigenesis. Our study provides evidence for SNX10 as a potential target for CRC treatment.

## Materials and methods

### Mice and CRC induction

SNX10 KO homozygous and littermate mice background in FVB were transferred from the Institute of Development Biology and Molecular Medicine, Fudan University, Shanghai, China. Male SNX10 KO homozygous mice and their WT littermate mice aged 8–10 weeks were injected intraperitoneally with a single dose (7.5 mg/kg) of AOM followed by three cycles of 2.5% (weight/volume) DSS given in the drinking water for 5 days^54^.

### Chemicals, reagents, and antibodies

Acetonitrile, methanol (HPLC grade), and dichloromethane were purchased from Sinopharm Chemical Reagent Company (Shanghai, China). Formic acid (HPLC grade) was purchased from Dima Technology (Lake Forest, CA, USA). And the standard chemicals for metabolite identification were obtained from Sigma-Aldrich (St. Louis, MO, USA) and Aladdin Industrial Corporation (Shanghai, China). Distilled water was filtered through a Milli-Q system (Millipore Corporation, Billerica, MA, USA). CQ (C6628), AOM (A5486), and EBSS (E2888) were purchased from Sigma-Aldrich. DSS (36–50 kDa, 0216011080) was purchased from MP Biomedicals (Santa Ana, CA, USA). SNX10 (sc-104657) and GAPDH (sc-47724) antibodies were the product of Santa Cruz Biotechnology (Dallas, TX, USA). Antibody against LAMP-2A (ab18528) was purchased from Abcam (Cambridge, UK). Antibody against β-actin (AA128) was from Beyotime Institute of Biotechnology (Haimeng, China). mTOR (2983) and p-mTOR (5536) antibodies were the product of Cell Signaling Technology (Boston, MA, USA).

### Tissue sample treatment and analysis by UHPLC-QTOF

Tissue samples were treated using protein precipitation method. Agilent UHPLC system (Infinity 1260, Agilent Technologies, Santa Clara, CA, USA) equipped with a UPLC ACQUITY HSS T3 column (ACQUITY HSS T3, 2.1 × 100 mm, 1.8 μm, Waters Corporation, Milford, MA, USA) were used for chromatographic separations. The UHPLC system was coupled to an Agilent 6520 electrospray ion source Accurate-Mass QTOF to obtain accurate mass time-of-flight data. For details, see Supplementary Experimental Procedures.

### Tissue sample treatment and analysis by GC-MS

Tissue samples were treated using methyl silane derivatization. MS spectra of the sample extracts were acquired on the GC-MS analytical system with a 7890B GC and 5977A quadrupole mass analyzer in full scan mode. For details, see Supplementary Experimental Procedures.

### Untargeted metabolomics data processing and metabolic pathway analysis

Raw mass spectra data were converted to mzDATA format files using Agilent MassHunter Qualitative Analysis (version B.06.00). Then the mzDATA files were uploaded to the XCMS Online (https://xcmsonline.scripps.edu) to preprocess to obtain the comma-separated value format peak intensity lists that contain *m*/*z*, retention time, and intensity of each peak. The further data statistic analysis was conducted by MetaboAnalyst 3.0 (http://www.metaboanalyst.ca) tool. The fully preprocessed data were analyzed with multivariate analysis methods of PCA and PLS-DA. Univariate analysis methods of FC analysis and *t*-test were also applied by the statistical analysis module of MetaboAnalyst.

Metabolic pathways and predicted metabolites in the pathways were analyzed utilizing mummichog 2.0 (http://mummichog.org) at the command line level. Mummichog is a program written in python for analyzing data from high-throughput, untargeted high-resolution LC-MS metabolomics, bypassing the tedious and challenging metabolite identification^55^. For GC-MS data, the feature ions with average variable importance in the projection (VIP) values of PLS-DA components higher than 1 (VIP > 1) were selected as discriminating features of further identification. The raw mass spectra data were imported to MassHunter for mass spectra deconvolution and metabolites identification by comparation of the *m*/*z* values with NIST databases. The compound with highest matching score was identified to the *m*/*z* value. For details, see Supplementary Experimental Procedures.

### Cell culture and transfection

NCM460, HCT116, Caco-2, and SW480 cells were purchased from the American Type Culture Collection (Manassas, VA, USA). Cells were cultured in Dulbecco’s modified Eagle’s (Gibco, Thermo Fisher Scientific, Waltham, MA, USA) supplemented with 10% fetal bovine serum (Biological Industries, Beit Haemek, Israel) at 37 °C under 5% (volume/volume) CO_2_ atmosphere. LAMP-2A siRNA was transfected into cells by Lipofectamine RNAi max (Invitrogen, Thermo Fisher Scientific, Waltham, MA, USA) for 72 h. The siRNA sequences are as follows: LAMP-2A siRNA: GGCAGGAGUACUUAUUCUAGU; nontargeting siRNA: AATTCTCCGAACGTGTCACGT.

### Immunofluorescence staining

After treatment, cells were fixed in 4% formaldehyde for 10 min at ambient temperature, followed by permeabilization and blocking with phosphate buffer solution (PBS) containing 10% normal goat serum and 0.1% saponin (Sigma-Aldrich). Cells were then incubated with primary antibodies for 2 h at ambient temperature. After washing three times with PBS, cells were incubated with appropriate Alexa Fluor-labeled secondary antibodies (1:500, Invitrogen, in blocking buffer) for 1 h at ambient temperature. The coverslips were mounted in Prolong Gold anti-fade reagent with 4′,6-diamidino-2-phenylindole (Invitrogen) and inspected with a confocal microscope (Zeiss 710, Zeiss Group, Oberkochen, Germany).

### Histological analysis

Mice colorectum were removed, fixed with formaldehyde solution, and sectioned routinely into 4–5 μm slabs, which were stained with hematoxylin and eosin, followed by analysis of tumor size using a light microscope.

### Cell proliferation assay

Cell proliferation was tested using BrdU ELISA kit according to the manufacturer’s instructions (Exalpha Biologicals, Shirley, MA, USA).

### Colony formation assay

WT and SXN10 KO cells were plated in six-well plates at approximately 200 cells/well in 2 ml of complete media. For SNX10 reintroduction group, SXN10 KO cells were infected with Adenovirus-vector or Adenovirus-SNX10 once a week. The assay should be stopped when the colonies are clearly visible even without looking under microscope. Cells then were stained with 0.1% crystal violet and number of colonies was counted.

### Western blot

Western blot analysis was performed as we described previously^56^.

### Cell targeted metabolomics by GC-MS

The medium was discarded and cells were washed with 10 ml ambient-temperature PBS (BasalMedia, China). Then, the plates were quenched by directly adding 15 ml of liquid nitrogen. The plates were transferred to a −80 °C freezer until extracted and assayed. For metabolites extraction, 1.2 ml of ice-cold methanol containing 20 μg/ml tridecanoic acid (Sigma-Aldrich) as ISTD was added to each plate and cells were detached from the surface with a disposable cell scraper (Crystalgen, China) and collected into a centrifuge tube for extraction. Cell lysates were then prepared with 30 min ultra-sonication. After centrifugation at 12 000 rounds/min for 10 min, supernatant was transferred and dried under the nitrogen stream at 40 °C. The subsequent derivatization procedures were as same as the untargeted metabolomics experiment. After derivatization, the supernatants were analyzed on the GC-MS analytical system with a 7890B GC and 5977A quadrupole mass analyzer (Agilent) in selected ion monitor mode. For details, see Supplementary Experimental Procedures.

### Pearson correlation coefficient computation

The Pearson correlation coefficient is calculated by the R language program. For details, see Supplementary Experimental Procedures.

### Statistics

Data are presented as mean ± SEM, Statistical analysis were performed by GraphPad Prism version 6.0. Unpaired two-tailed Student’s *t*-test or one-way analysis of variance was used to compare the mean values of the groups. Statistical significance was accepted at *P* < 0.05.

## Electronic supplementary material

Supplementary Materials
