# Supplementary material for: Sorting nexin 10 controls mTOR activation through regulating amino-acid metabolism in colorectal cancer
Source: Cell Death Dis. 2018 Jun 4;9(6):666. doi: 10.1038/s41419-018-0719-2 (PMC5986761; doi:10.1038/s41419-018-0719-2)
Supplement: Supplementary file 1 — Supplementary Materials [file 41419_2018_719_MOESM1_ESM.docx]

**Supplemental Information**

**Sorting nexin 10 controls mTOR activation through regulating amino acid metabolism in colorectal cancer**

Yunchen Le, Sulin Zhang, Jiahui Ni, Yan You, Kejing Luo, Yunqiu Yu, Xiaoyan Shen

**Supplemental Data**

Supplementary Figures


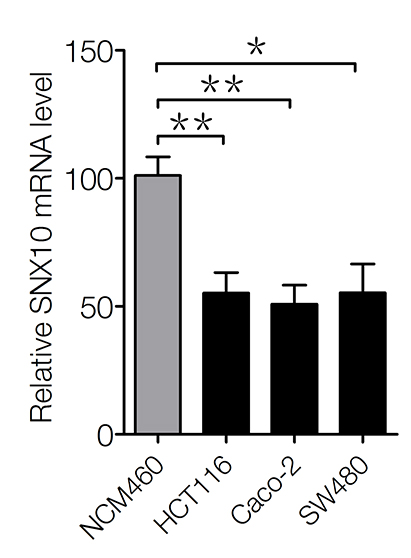


**Fig. S1 SNX10 mRNA levels in human normal colonic epithelial cell line (NCM460) and three human colonic cancer cell lines (HCT116, Caco-2, SW480) were measured by RT-qPCR.**


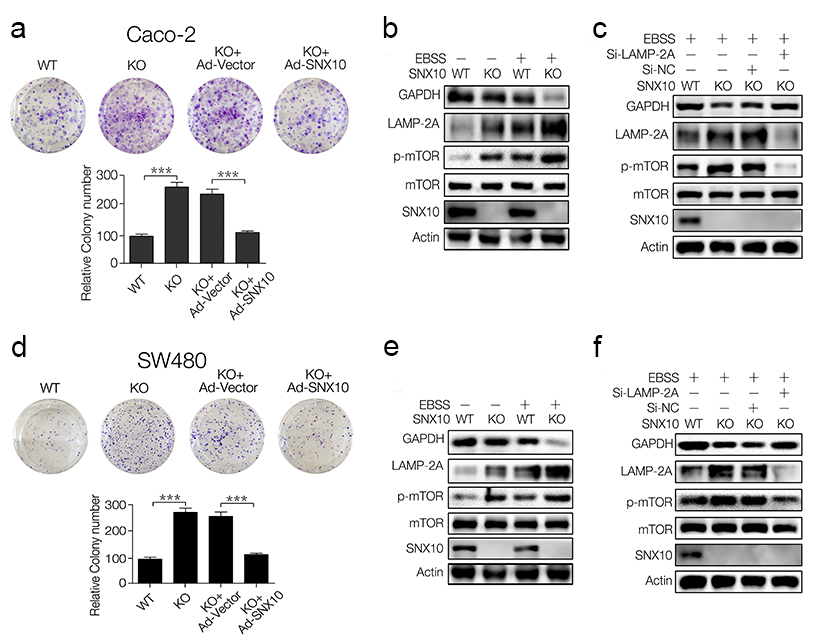


**Fig. S2 The colony-forming ability and representative Western blots of Caco-2 and SW480 cells.**

**a** The colony-forming ability of WT, SXN10 KO Caco-2 cells and SXN10 KO Caco-2 cells infected with Ad-vector or Ad-SNX10. **b** Representative Western blots showed the expression of indicated proteins in WT and SNX10 KO Caco-2 cells with or without the treatment of EBSS for 24 h. **c** WT, SNX10 KO Caco-2 cells and SNX10 KO Caco-2 cells transfected with nontargeting siRNA (Si-NC) or LAMP-2A siRNA (Si-LAMP-2A) were treated with EBSS for 24 h, the total proteins were isolated and immunobloted with indicated antibodies. **d** The colony-forming ability of WT, SXN10 KO SW480 cells and SXN10 KO SW480 cells infected with Ad-vector or Ad-SNX10. **e** Representative Western blots showed the expression of indicated proteins in WT and SNX10 KO SW480 cells with or without the treatment of EBSS for 24 h. **f** WT, SNX10 KO SW480 cells and SNX10 KO SW480 cells transfected with nontargeting siRNA (Si-NC) or LAMP-2A siRNA (Si-LAMP-2A) were treated with EBSS for 24 h, the total proteins were isolated and immunobloted with indicated antibodies.


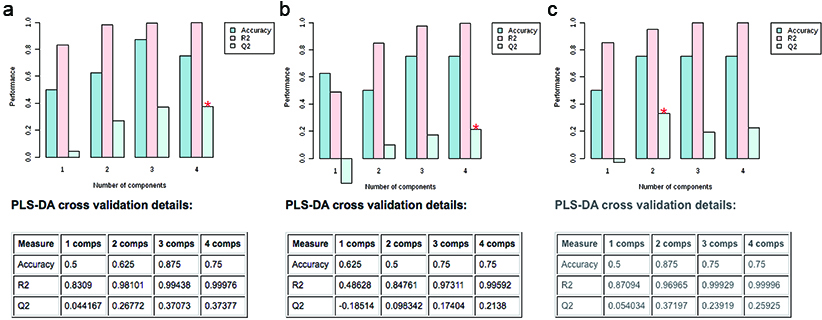


**Fig. S3 Cross validation bar graph is used to determine the optimal number of components needed to build the PLS-DA model. There are three common performance measures—the sum of squares captured by the model (R2), the cross-validated R2 (also known as Q2), and the prediction accuracy (Accuracy).** **a** The cross validation bar graph and details of untargeted metabolomics data in positive ion mode UHPLC-QTOF. **b** The cross validation bar graph and details of untargeted metabolomics data in negative ion mode UHPLC-QTOF. **c** The cross validation bar graph and details of untargeted metabolomics data in GC-MS.


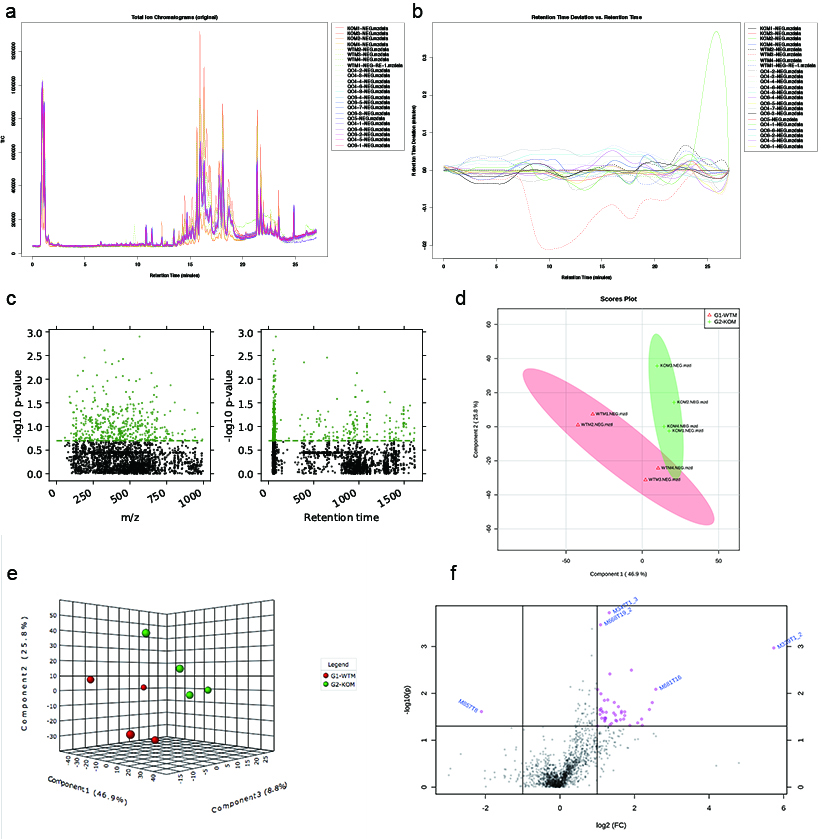


**Fig. S4 Untargeted metabolomics and multivariate statistical analysis of CRC male FVB mice tumor tissues using UHPLC-QTOF in negative ion mode.** **a** Total ion chromatograms of WT (WTM), SNX10 KO (KOM) and quality control samples (QC). **b** Retention time deviation of all samples. **c** Manhattan map shows the distribution of significant features (*P* < 0.05, green dots) in m/z and retention time. **d** 2D scores plot between the selected PCs of PLS-DA model. **e** 3D scores plot between the selected PCs of PCA model. **f** Volcano plot with fold change threshold and t-tests threshold to select the important features.


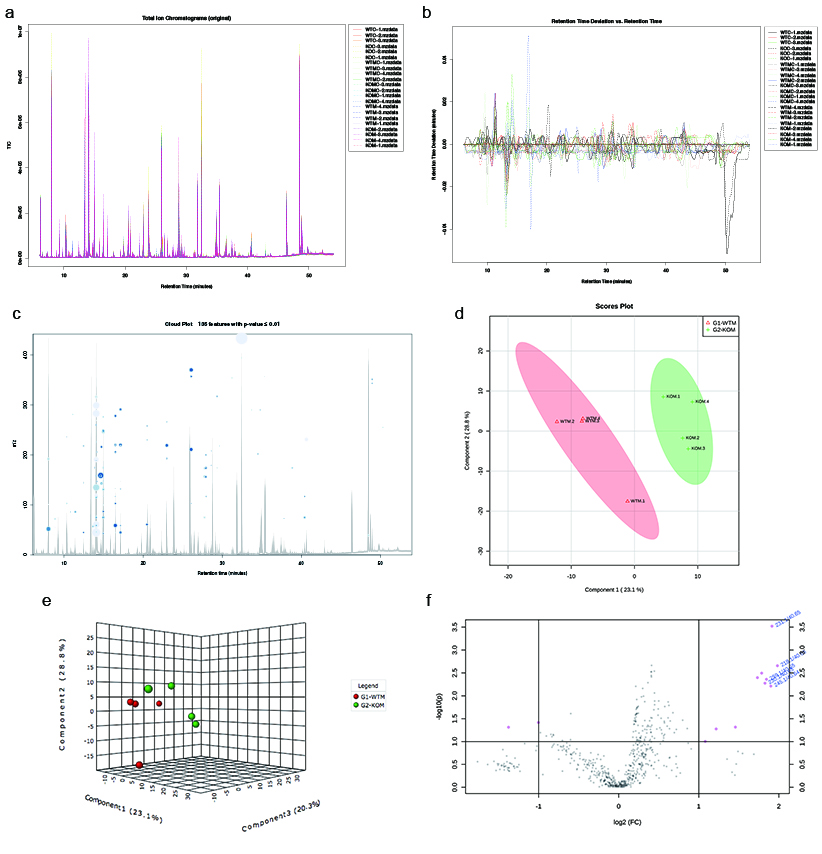


**Fig. S5 Untargeted metabolomics and multivariate statistical analysis of CRC male FVB mice tumor tissues using GC-MS**. **a** Total ion chromatograms of WT (WTM), SNX10 KO (KOM) and quality control samples (QC). **b** Retention time deviation of all samples. **c** Cloud map shows the distribution of significant features (*P* < 0.05, blue dots) in m/z and retention time. **d** 2D scores plot between the selected PCs of PLS-DA model. **e** 3D scores plot between the selected PCs of PCA model. **f** Volcano plot with fold change threshold and t-tests threshold to select the important features.

**
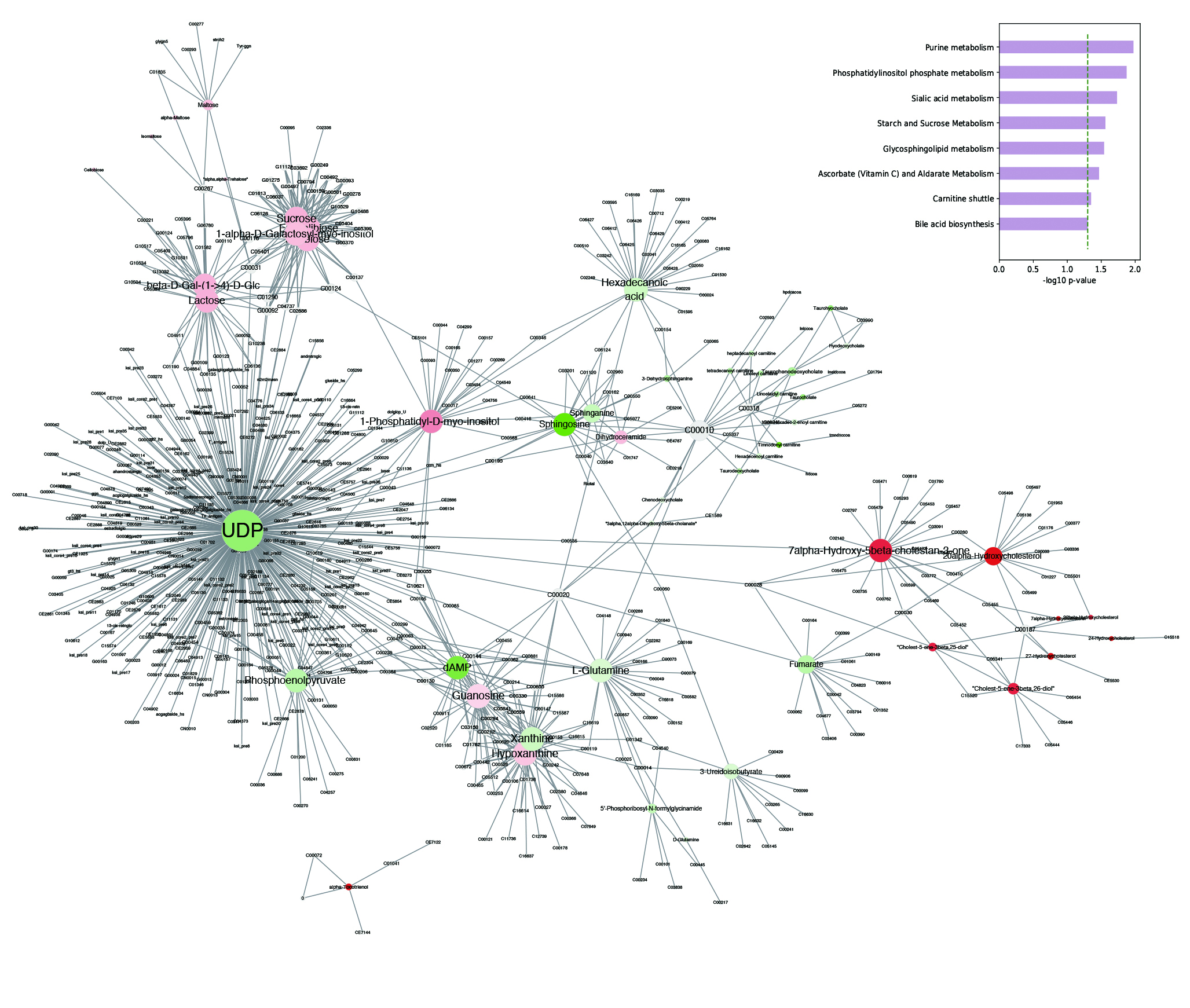
**

**Fig. S6 Metabolic pathways networks based on the untargeted metabolomics using UHPLC-QTOF in positive ion mode.**

**Supplementary Tables**

**Table S1** Metabolites identified by untargeted metabolomics in positive ion mode UHPLC-QTOF

| **Compound** | **Input m/z** | **Retention time (Seconds)** | **ion** | **log2(FC)** |
| --- | --- | --- | --- | --- |
| 3α,12α-Dihydroxy-5β-cholanate | 392.2899129 | 1513.32 | M[1+] | -0.17 |
| 5'-Phosphoribosyl-N-formylglycinamide | 337.0417801 | 45.83 | M+Na[1+] | -0.45 |
| Linoelaidyl carnitine | 424.3436272 | 982.37 | M+H[1+] | -0.65 |
| Timnodonyl carnitine | 468.3080329 | 513.265 | M+Na[1+] | -1.99 |
| Hypoxanthine | 205.033957 | 69.745 | M+HCOONa[1+] | 0.6 |
| Taurochenodeoxycholate | 522.288885 | 881.785 | M+Na[1+] | -0.62 |
| Phosphoenolpyruvate | 190.9718684 | 651.29 | M+Na[1+] | -0.52 |
| α-Tocotrienol | 425.3428066 | 1231.95 | M+H[1+] | 3.15 |
| Hexadecanoic acid | 257.2495189 | 1241.89 | M+H[1+] | -0.36 |
| Sphinganine | 302.3057296 | 913.66 | M+H[1+] | -0.43 |
| Fumarate | 117.0180844 | 47.595 | M+H[1+] | -0.55 |
| Dihydroceramide | 330.2994945 | 878.04 | M+H[1+] | 0.72 |
| Melibiose | 343.1200864 | 754.74 | M+H[1+] | 0.81 |
| heptadecanoyl carnitine | 414.3594016 | 1205.095 | M+H[1+] | -0.39 |
| Linoelaidyl carnitine | 446.3274324 | 1405.98 | M+Na[1+] | -0.57 |
| dAMP | 332.0723554 | 981.44 | M+H[1+] | -1.34 |
| Taurochenodeoxycholate | 500.3029626 | 755.83 | M+H[1+] | -0.62 |
| 3-Ureidoisobutyrate | 147.0768351 | 56.34 | M+H[1+] | -0.27 |
| α-Tocotrienol | 425.3422179 | 1252.86 | M+H[1+] | 3.88 |
| Xanthine | 153.0415792 | 68.84 | M+H[1+] | -0.37 |
| Taurohyocholate | 517.3053588 | 401.69 | M(C13)+H[1+] | -0.95 |
| Hyodeoxycholate | 392.2899129 | 1513.32 | M[1+] | -0.17 |
| trans-Hexadec-2-enoyl carnitine | 420.3062423 | 1194.3 | M+Na[1+] | -0.27 |
| α-Tocotrienol | 447.3205926 | 1435.79 | M+Na[1+] | -4.46 |
| Hyodeoxycholate | 393.3018665 | 1024.255 | M+H[1+] | -0.57 |
| Taurochenodeoxycholate | 568.2889188 | 858.54 | M+HCOONa[1+] | -0.88 |
| 1-Phosphatidyl-D-myo-inositol | 413.0456978 | 69.935 | M+Na[1+] | 1.45 |
| Sphingosine | 322.2745304 | 1183.24 | M+Na[1+] | -2.38 |
| UDP | 426.989485 | 388.62 | M+Na[1+] | -0.95 |
| 7α-Hydroxycholesterol | 425.3428066 | 1231.95 | M+Na[1+] | 3.15 |
| Timnodonyl carnitine | 446.3274324 | 1405.98 | M+H[1+] | -0.57 |
| tetradecanoyl carnitine | 372.3090954 | 1225.52 | M+H[1+] | -0.61 |
| 7α-Hydroxycholesterol | 425.3422179 | 1252.86 | M+Na[1+] | 3.88 |
| Guanosine | 306.0809848 | 70.175 | M+Na[1+] | 0.43 |

*Abbreviation: dAMP, deoxyadenosine monophosphate; UDP, uridine diphosphate*

**Table S2** Metabolites identified by untargeted metabolomics in negative ion mode UHPLC-QTOF

| **Compound** | **Input m/z** | **Retention time (seconds)** | **ion** | **log2(FC)** |
| --- | --- | --- | --- | --- |
| D-Glucuronate | 229.0103833 | 57.71 | M+Cl[-] | 0.49 |
| (R)-S-Lactoylglutathione | 394.0943762 | 70.68 | M-H+O[-] | 0.37 |
| (S)-Malate | 133.0130612 | 62.74 | M-H[-] | 0.63 |
| 1-(5'-Phosphoribosyl)-5-amino-4-(N-succinocarboxamide)-imidazole | 435.0532373 | 60.78 | M-H2O-H[-] | 1.33 |
| 1-(5'-Phosphoribosyl)-5-formamido-4-imidazolecarboxamide | 366.0505715 | 69.72 | M(C13)-H[-] | 0.71 |
| 2,3-Diketo-L-gulonate | 191.0182922 | 68.64 | M-H[-] | 1 |
| 3-Hydroxypropanoate | 89.0238407 | 72.53 | M-H[-] | 0.71 |
| 6-Phospho-2-dehydro-D-gluconate | 272.9991918 | 56.515 | M-H[-] | 0.65 |
| 6-Phospho-D-gluconate | 275.0169603 | 62.695 | M-H[-] | 1.89 |
| Acetate | 119.0347872 | 55.695 | M+CH3COO[-] | -0.42 |
| Acetone | 57.03412188 | 58.765 | M-H[-] | 0.58 |
| Acetyl phosphate | 138.978917 | 56.83 | M-H[-] | 0.67 |
| Adenosine 3',5'-bisphosphate | 408.0118807 | 62.205 | M-H2O-H[-] | 0.5 |
| Allantoate | 157.0356896 | 58.77 | M-H2O-H[-] | -0.7 |
| Allantoin | 157.0356896 | 58.77 | M-H[-] | -0.7 |
| α-D-Galactose | 89.0238407 | 72.53 | M-2H[2-] | 0.71 |
| α-D-Glucose 6-phosphate | 275.0169603 | 62.695 | M-H+O[-] | 1.89 |
| α-Tocotrienol | 423.3228066 | 1472.79 | M-H[-] | 0.72 |
| AMP | 346.0533712 | 60.67 | M-H[-] | 0.76 |
| CMP-N-acetylneuraminate | 613.137034 | 59.74 | M-H[-] | 0.43 |
| D-Galactose | 89.0238407 | 72.53 | M-2H[2-] | 0.71 |
| D-Glucarate | 191.0182922 | 68.64 | M-H2O-H[-] | 1 |
| D-Glucuronolactone | 191.0182922 | 68.64 | M-H+O[-] | 1 |
| D-Glyceraldehyde | 89.0238407 | 72.53 | M-H[-] | 0.71 |
| D-Glyceraldehyde 3-phosphate | 229.0103833 | 57.71 | M+CH3COO[-] | 0.49 |
| Dihydrolipoate | 267.0722011 | 70.68 | M+CH3COO[-] | 0.76 |
| D-myo-Inositol 1,3-bisphosphate | 168.989331 | 57.72 | M-2H[2-] | 0.55 |
| D-myo-Inositol 1,3-bisphosphate | 338.987072 | 83.02 | M-H[-] | 6 |
| D-Ribulose 5-phosphate | 229.0103833 | 57.71 | M-H[-] | 0.49 |
| D-Sedoheptulose 1,7-bisphosphate | 369.0016375 | 70.695 | M-H[-] | 2.69 |
| D-Sedoheptulose 7-phosphate | 327.0072637 | 51.7 | M+Cl37[-] | 0.77 |
| D-Serine | 140.0101746 | 54.72 | M+Cl[-] | 0.84 |
| D-Xylulose 5-phosphate | 275.0169603 | 62.695 | M+HCOO[-] | 1.89 |
| FAD | 784.1445636 | 389.49 | M-H[-] | 0.94 |
| Glutathione disulfide | 611.1399818 | 61.74 | M-H[-] | 0.66 |
| GMP | 362.0483732 | 62.665 | M-H[-] | 1.07 |
| Guanosine | 318.0596158 | 69.73 | M+Cl[-] | 1.14 |
| Hypoxanthine | 135.0299961 | 67.69 | M-H[-] | 0.29 |
| IMP | 347.0389698 | 62.48 | M-H[-] | 1.47 |
| Inosine | 267.0722011 | 70.68 | M-H[-] | 0.76 |
| L-Erythrulose | 135.0299961 | 67.69 | M-H+O[-] | 0.29 |
| L-Fucose 1-phosphate | 243.0255897 | 55.83 | M-H[-] | 1.07 |
| N-Acetylneuraminate | 308.0966395 | 57.74 | M-H[-] | 0.59 |
| Propane-1,2-diol | 57.03412188 | 58.765 | M-H2O-H[-] | 0.58 |
| Pyruvate | 133.0130612 | 62.74 | M+HCOO[-] | 0.63 |
| Sedoheptulose 7-phosphate | 327.0072637 | 51.7 | M+Cl37[-] | 0.77 |
| sorbitol 3-phosphate | 259.0210019 | 56.79 | M-H[-] | 0.53 |
| Threonate | 135.0299961 | 67.69 | M-H[-] | 0.29 |
| UDP-D-galactose | 565.043838 | 80.125 | M-H[-] | 1.72 |
| UDP-N-acetyl-D-galactosamine | 606.0742217 | 60.77 | M-H[-] | 1.28 |

*Abbreviation: AMP, adenosine monophosphate; CMP, cytidine monophosphate; FAD, Flavin adenine dinucleotide; GMP, guanosine monophosphate; IMP, Inosine monophosphate; UDP, uridine diphosphate*

**Table S3** Metabolites identified by untargeted metabolomics using GC-MS

| **Compound** | **m/z** | **Retention time (minuets)** | **VIP** | **Log2(FC)** |
| --- | --- | --- | --- | --- |
| inosine | 231.1 | 40.65 | 3.104575 | -1.562293406 |
| inositol | 354.1 | 31.24 | 3.478475 | 1.655801964 |
| uridine | 217.1 | 38.61 | 2.316275 | -1.072705697 |
| α-D-Galactopyranoside | 247.1 | 48.75 | 1.01595 | -0.231873676 |
| serine | 59 | 16.5 | 1.459775 | 1.175265607 |
| cholesterol | 213.1 | 48.5 | 2.89655 | -0.735910224 |
| tyrosine | 208.1 | 28.63 | 1.122225 | 0.983560228 |
| L-threonine | 180 | 14.69 | 3.351525 | 1.584243012 |
| L-proline | 216.1 | 14.73 | 2.93125 | 1.479786403 |
| 1,5-dimethylbarbituric acid | 317.2 | 29.36 | 2.3057 | 1.105095954 |
| L-leucine | 260.1 | 14.06 | 3.8224 | 1.793570202 |
| 1,4-butanediamine | 175.1 | 27.94 | 1.17135 | 0.963354219 |
| L-phenylalanine | 266.1 | 23 | 1.2762 | 0.989268894 |
| urea | 75 | 13.38 | 1.9508 | 1.909949546 |
| L-valine | 55 | 8.87 | 1.249875 | 1.092645198 |
| D-mannose | 315.1 | 36.34 | 1.249825 | 0.857283222 |
| Maltose | 204.1 | 42.96 | 3.914925 | -0.949169691 |
| N,N,N',O-Tetra-tryptophane | 202.1 | 34.52 | 1.134375 | 0.898143508 |
| phosphoric acid | 242 | 11.4 | 1.952475 | -1.310339869 |
| 2-ketoglutaric acid | 157 | 27.77 | 1.07705 | -0.410232113 |
| allonic acid | 87 | 29.56 | 2.0227 | -0.785145508 |
| D-fructose | 103 | 28.3 | 1.122525 | 0.737400634 |
| D-(+)-talose | 73.1 | 28.79 | 1.213515 | -0.387142529 |
| D-(-)-lactic acid | 73 | 8.03 | 2.88385 | 0.207386459 |

**Table S4** Relative response to the internal standard of analytes in CRC cells measured using GC-MS (related to Fig. 6b)

|  | CRC-CELLS-1-1-1218.D | CRC-CELLS-1-2-1218.D | CRC-CELLS-1-3-1218.D | CRC-CELLS-2-1-1218.D | CRC-CELLS-2-2-1218.D | CRC-CELLS-2-3-1218.D | CRC-CELLS-3-1-1218.D | CRC-CELLS-3-2-1218.D | CRC-CELLS-3-3-1218.D | CRC-CELLS-4-1-1218.D | CRC-CELLS-4-2-1218.D | CRC-CELLS-4-3-1218.D |
| --- | --- | --- | --- | --- | --- | --- | --- | --- | --- | --- | --- | --- |
| Ala | 1.242 | 1.187 | 1.559 | 1.562 | 1.458 | 1.553 | 1.596 | 1.538 | 1.607 | 1.724 | 1.743 | 1.734 |
| Val | 1.650 | 1.635 | 1.735 | 2.666 | 2.301 | 2.381 | 2.345 | 2.331 | 2.338 | 2.182 | 2.284 | 2.216 |
| Leu | 0.933 | 0.867 | 0.861 | 1.727 | 1.384 | 1.691 | 1.398 | 1.363 | 1.381 | 1.125 | 1.047 | 1.105 |
| Ile | 2.045 | 1.927 | 1.936 | 3.175 | 2.848 | 3.263 | 2.907 | 2.849 | 2.868 | 2.799 | 2.731 | 2.745 |
| Pro | 0.427 | 0.394 | 0.395 | 0.952 | 0.718 | 0.953 | 0.751 | 0.731 | 0.677 | 0.620 | 0.578 | 0.563 |
| Gly | 4.399 | 4.175 | 4.222 | 6.181 | 5.776 | 5.838 | 5.788 | 5.547 | 5.397 | 4.873 | 4.797 | 4.786 |
| Ser | 0.311 | 0.302 | 0.330 | 0.870 | 0.854 | 0.859 | 0.532 | 0.548 | 0.545 | 0.438 | 0.447 | 0.446 |
| Thr | 2.038 | 1.911 | 1.951 | 3.591 | 3.408 | 3.337 | 3.026 | 2.915 | 2.846 | 2.739 | 2.716 | 2.616 |
| Asp | 0.403 | 0.443 | 0.554 | 1.544 | 1.535 | 1.511 | 1.479 | 1.447 | 1.458 | 1.230 | 1.252 | 1.262 |
| Met | 0.744 | 0.780 | 0.847 | 1.293 | 1.279 | 1.242 | 1.460 | 1.426 | 1.413 | 1.331 | 1.366 | 1.355 |
| Glu | 3.094 | 2.967 | 2.885 | 2.671 | 2.663 | 2.691 | 3.007 | 2.994 | 2.988 | 3.025 | 3.049 | 3.070 |
| Phe | 2.183 | 2.163 | 2.239 | 2.084 | 2.057 | 2.083 | 2.588 | 2.539 | 2.538 | 2.677 | 2.686 | 2.693 |
| Asn | 0.088 | 0.073 | 0.091 | 0.443 | 0.504 | 0.444 | 0.438 | 0.419 | 0.411 | 0.371 | 0.381 | 0.387 |
| Arg | 1.101 | 1.057 | 1.041 | 1.000 | 1.019 | 0.975 | 1.165 | 1.141 | 1.108 | 1.166 | 1.139 | 1.137 |

*Abbreviation: Ala, alanine; Val, valine; Leu, leucine; Ile, isoleucine; Pro, proline; Gly, glycine; Ser, serine; Thr, threonine; Asp, aspatic acid; Met, methionine; Glu, glutamic acid; Phe, phenyalanine; Asn, asparagine; Arg, arginine*

**Table S5** Pearson correlation coefficient of amino acids, LAMP-2A and p-mTOR (related to Fig. 6d)

|  | Ala | Val | Leu | Ile | Pro | Gly | Ser | Thr | Asp | Met | Glu | Phe | Asn | Arg | LAMP2A | p-mTOR |
| --- | --- | --- | --- | --- | --- | --- | --- | --- | --- | --- | --- | --- | --- | --- | --- | --- |
| Ala | 1.00 | 0.63 | 0.27 | 0.59 | 0.35 | 0.29 | 0.19 | 0.42 | 0.63 | 0.77 | 0.09 | 0.68 | 0.62 | 0.37 | -0.09 | 0.49 |
| Val | 0.63 | 1.00 | 0.87 | 0.96 | 0.90 | 0.90 | 0.79 | 0.94 | 0.96 | 0.88 | -0.43 | 0.18 | 0.94 | -0.05 | 0.59 | 0.94 |
| Leu | 0.27 | 0.87 | 1.00 | 0.90 | 0.99 | 0.97 | 0.92 | 0.93 | 0.84 | 0.63 | -0.66 | -0.22 | 0.80 | -0.38 | 0.82 | 0.89 |
| Ile | 0.59 | 0.96 | 0.90 | 1.00 | 0.93 | 0.90 | 0.82 | 0.94 | 0.96 | 0.86 | -0.43 | 0.15 | 0.95 | -0.08 | 0.59 | 0.94 |
| Pro | 0.35 | 0.90 | 0.99 | 0.93 | 1.00 | 0.96 | 0.91 | 0.95 | 0.86 | 0.67 | -0.63 | -0.16 | 0.83 | -0.33 | 0.75 | 0.89 |
| Gly | 0.29 | 0.90 | 0.97 | 0.90 | 0.96 | 1.00 | 0.90 | 0.96 | 0.89 | 0.71 | -0.61 | -0.15 | 0.86 | -0.26 | 0.85 | 0.94 |
| Ser | 0.19 | 0.79 | 0.92 | 0.82 | 0.91 | 0.90 | 1.00 | 0.93 | 0.78 | 0.50 | -0.85 | -0.42 | 0.77 | -0.58 | 0.74 | 0.80 |
| Thr | 0.42 | 0.94 | 0.93 | 0.94 | 0.95 | 0.96 | 0.93 | 1.00 | 0.93 | 0.75 | -0.64 | -0.10 | 0.93 | -0.26 | 0.70 | 0.93 |
| Asp | 0.63 | 0.96 | 0.84 | 0.96 | 0.86 | 0.89 | 0.78 | 0.93 | 1.00 | 0.93 | -0.40 | 0.23 | 0.99 | 0.01 | 0.62 | 0.98 |
| Met | 0.77 | 0.88 | 0.63 | 0.86 | 0.67 | 0.71 | 0.50 | 0.75 | 0.93 | 1.00 | -0.06 | 0.56 | 0.92 | 0.35 | 0.41 | 0.89 |
| Glu | 0.09 | -0.43 | -0.66 | -0.43 | -0.63 | -0.61 | -0.85 | -0.64 | -0.40 | -0.06 | 1.00 | 0.74 | -0.38 | 0.87 | -0.60 | -0.42 |
| Phe | 0.68 | 0.18 | -0.22 | 0.15 | -0.16 | -0.15 | -0.42 | -0.10 | 0.23 | 0.56 | 0.74 | 1.00 | 0.24 | 0.90 | -0.37 | 0.14 |
| Asn | 0.62 | 0.94 | 0.80 | 0.95 | 0.83 | 0.86 | 0.77 | 0.93 | 0.99 | 0.92 | -0.38 | 0.24 | 1.00 | 0.04 | 0.57 | 0.96 |
| Arg | 0.37 | -0.05 | -0.38 | -0.08 | -0.33 | -0.26 | -0.58 | -0.26 | 0.01 | 0.35 | 0.87 | 0.90 | 0.04 | 1.00 | -0.38 | -0.03 |
| LAMP2A | -0.09 | 0.59 | 0.82 | 0.59 | 0.75 | 0.85 | 0.74 | 0.70 | 0.62 | 0.41 | -0.60 | -0.37 | 0.57 | -0.38 | 1.00 | 0.75 |
| p-mTOR | 0.49 | 0.94 | 0.89 | 0.94 | 0.89 | 0.94 | 0.80 | 0.93 | 0.98 | 0.89 | -0.42 | 0.14 | 0.96 | -0.03 | 0.75 | 1.00 |

*Abbreviation: Ala, alanine; Val, valine; Leu, leucine; Ile, isoleucine; Pro, proline; Gly, glycine; Ser, serine; Thr, threonine; Asp, aspatic acid; Met, methionine; Glu, glutamic acid; Phe, phenyalanine; Asn, asparagine; Arg, arginine*

**Supplemental Experimental Procedures**

**Tissue samples treatment for UHPLC-QTOF analysis**

Tissue samples were thawed on dry ice, weighted and ultrasound in -20°C cold acetonitrile and water (1:1, v/v) (50μL to every 1mg of tissue) for 10 min. Then samples were homogenized with washed stainless beads on an electric tissue homogenizer, using a frequency of 60 Hz for 120 s, twice for each sample. After that stainless beads were removed. Samples were centrifuged at 12 000 rpm for10 min. After another centrifugation at 12 000 rpm for 10 min, the supernatant was transfer to UHPLC-QTOF full scan analysis.

**UHPLC-QTOF data acquisition:**

A 5 μL aliquot of the supernatant were injected onto an Agilent UHPLC system (Infinity 1260) equipped with a UPLC ACQUITY HSS T3 column (ACQUITY HSS T3, 2.1×100 mm, 1.8 μm, Waters Corporation, Milford, MA, U.S.A) at a column temperature of 35 °C. The UHPLC system was coupled to an Agilent 6520 electrospray ion source Accurate-Mass QTOF (Agilent Technologies, Santa Clara, CA, USA). The flow rate was 0.3 mL/min and the mobile phase were water consisted of 0.1% formic acid solution (A) and acetonitrile containing 0.1% formic acid (B), respectively. The linear gradient conditions were set as follows: time (t) = 0-1 min, hold B at 1%; t = 1-5 min, increase B from 1 to 40%; t = 5-8 min, increase B from 40 to 50%; t = 8-10 min, increase B from 50 to 65%; t = 10-16 min, increase B from 65 to 76%; t = 16-20 min, increase B from 76 to 100%; t = 20-25 min hold B at 100%; t = 25-27 min, decrease B from 100 to 1%. Data were collected by operating the mass spectrometer in positive and negative electrospray ionizations in centroid and profile mode, in full-scan range from 50 to 1000 m/z. Nitrogen was used as both cone gas and desolvation gas with a flow of 10 L/min). Nebulizer pressure was set at 40 psi. The desolvation temperature and capillary voltages were set at 350℃and 3500 V, respectively.

**Tissue samples treatment for GC-MS analysis**

Tissue samples were thawed on dry ice, weighted and ultrasound in -20°C cold tridecanoic acid (40 μg/mL in methanol solution) (50μL to every 1mg of tissue) for 10 min. Tridecanoic acid was the internal standard. Then samples were homogenized with washed stainless beads on an electric tissue homogenizer, using a frequency of 60 Hz for 120 s, twice for each sample. After that stainless beads were removed. Samples were centrifuged at 12 000 rpm for 10 min. The supernatants were evaporated to dryness by concentrator under the nitrogen stream at 40°C. 50 μL MOX (15mg/mL in pyridine solution) was added to the dried sample and vortexed for 5 min. samples were incubated at 40°C oven for about 1 h to oximation. Silylation reaction was performed by adding 40 μl MSTFA per sample and vortexed for 5 min, then incubated in the oven for 1h at 40°C. Samples were centrifuged at 12 000 rpm for 5 min at 4°C. 30 μL supernatant were transfer to GC glass vial equipped with a 200 μL insert for GC-MS analysis.

**GC-MS full scan data acquisition**

MS spectra of the sample extracts were acquired on the GC-MS analytical system with a 7890B GC and 5977A quadrupole mass analyzer (Agilent technologies) in full scan mode. HP-5 MS capillary column (30m×250μm×0.25μm) was applied to metabolites chromatographic separation. The sample injection volume was 1 μL with 20:1 split ratio and helium flow rate was adjusted to 1.2 mL/min. The injection port, interface and MS detector temperatures were set at 300°C, 280°C and 230°C, respectively. The column temperature was initially set at 70°C, kept for 3 min, and then increase to 300°C (5°C/min), and finally held for 5 min. Gain factor was set at 2.0, and electron impact (70 eV) was applied as the ionization mode. Mass signal acquisition (33-600, m/z) was performed after 6.0 min of solvent delay.

**Python programming for metabolic pathways analysis and metabolites identification, related to Figure S4 and Table S1**

leyunchendeMacBook-Air:~ leyunchen$ mummichog -f lcms-pos-mum.txt -o lcms-pos-output -n mouse

mummichog version 2.0.4-beta-20170719

Read 8567 features as reference list.

Automatically choosing (p < 0.050000) as significant cutoff.

Using 835 features (p < 0.050000) as significant list.

Got 1326 cpd2mzFeatures

Got 2741 ListOfEmpiricalCompounds

Got 1416 merged ListOfEmpiricalCompounds

Got 640 final ListOfEmpiricalCompounds

Pathway Analysis...

Query number of significant compounds = 73 compounds

Resampling, 100 permutations to estimate background ...

1 2 3 4 5 6 7 8 9 10 11 12 13 14 15 16 17 18 19 20 21 22 23 24 25 26 27 28 29 30 31 32 33 34 35 36 37 38 39 40 41 42 43 44 45 46 47 48 49 50 51 52 53 54 55 56 57 58 59 60 61 62 63 64 65 66 67 68 69 70 71 72 73 74 75 76 77 78 79 80 81 82 83 84 85 86 87 88 89 90 91 92 93 94 95 96 97 98 99 100

Pathway background is estimated on 11900 random pathway values

Modular Analysis, using 100 permutations ...

1 2 3 4 5 6 7 8 9 10 11 12 13 14 15 16 17 18 19 20 21 22 23 24 25 26 27 28 29 30 31 32 33 34 35 36 37 38 39 40 41 42 43 44 45 46 47 48 49 50 51 52 53 54 55 56 57 58 59 60 61 62 63 64 65 66 67 68 69 70 71 72 73 74 75 76 77 78 79 80 81 82 83 84 85 86 87 88 89 90 91 92 93 94 95 96 97 98 99 100

Null distribution is estimated on 1329 random modules

User data yield 10 network modules

Activity network was connected in 2 steps.

**GC-MS selective ion mode (SIM) data acquisition**

MS spectra of the sample extracts were acquired on the GC-MS analytical system with a 7890B GC and 5977A quadrupole mass analyzer (Agilent technologies) in selective ion mode. HP-5 MS capillary column (30m×250μm×0.25μm) was applied to metabolites chromatographic separation. The sample injection volume was 1 μL with 10:1 split ratio and helium flow rate was adjusted to 1.2 mL/min. The injection port, interface and MS detector temperatures were set at 300°C, 280°C and 230°C, respectively. The column temperature was initially set at 70°C, kept for 3 min, and then increase to 300°C (10°C/min), and finally held for 5 min. Gain factor was set at 2.0, and electron impact (70 eV) was applied as the ionization mode. Mass signal acquisition was performed after 6.0 min of solvent delay. We set 8 SIM groups according to the retention time and the fragment ions m/z of the analytes. SIM groups information: group 1 (scan time is 6-9 min, selective ions are 73.00, 115.00, 116.00, 117.00, 147.00, 174.00, 190.00, 91.00), group 2 (scan time is 9-10.5 min, selective ions are 73.00, 144.00, 147.00, 158.00, 189.00, 218.00), group 3 (scan time is 10.5-11 min, selective ions are 73.00, 142.00, 47.00, 158.00, 174.00, 218.00, 247.00, 248.00), group 4 (scan time is 11-12 min, selective ions are 73.00, 117.00, 147.00, 204.00, 218.00, 245.00), group 5 (scan time is 12 -14 min, selective ions are 73.00, 100.00, 128.00, 133.00, 147.00, 176.00, 218.00, 232.00, 233.00, 245.00), group 6 (scan time is 14-16 min, selective ions are 73.00, 116.00, 128.00, 132.00, 147.00, 192.00, 198.00, 218.00, 231.00, 246.00), group 7 (scan time is 16-16.9 min, selective ions are 73.00, 117.00, 147.00, 156.00, 229.00, 245.00, 271.00, 375.00), group 8 (scan time is 16.9-31 min, 73.00, 142.00, 147.00, 157.00, 174.00, 256.00, 273.00, 363.00).

**R programming for Pearson correlation coefficient calculation, related to Figure 6D and Table S5**

AA <- read.csv('AA_cor.csv', check.names = FALSE)

cor_AA = cor(AA)

cor_AA

write.csv(cor_AA, file = "cor_AA.csv")

library(corrplot)

corrplot(cor_AA, type="upper", order="original", tl.col="black", tl.srt=45, tl.cex = 1.5)

library(PerformanceAnalytics)

chart.Correlation(ampk_pos,histogram = TRUE,pch=19)
